# Modulation of mitochondrial quality control in sepsis by traditional Chinese medicine: mechanistic insights and translational perspectives

**DOI:** 10.3389/fphar.2026.1865750

**Published:** 2026-09-08

**Authors:** Maoxiao Chang, Dingji Hu, Kexin Zhang, Suxia Liu, Haoyue Xue, Yike Zhu, Shanshan Chen, Lingjuan Liu, Changde Wu, Haoya Fu, Jing Wu, Shixin Yuan, Jianfeng Xie, Songqiao Liu, Yi Yang, Haibo Qiu

**Affiliations:** 1 Jiangsu Provincial Key Laboratory of Critical Care Medicine, Department of Critical Care Medicine, Trauma Center, Zhongda Hospital, School of Medicine, Southeast University, Nanjing, Jiangsu, China; 2 The First People’s Hospital of Lianyungang, The Affiliated Lianyungang Hospital of Xuzhou Medical University, The Lianyungang Clinical College of Nanjing Medical University, Lianyungang, Jiangsu, China

**Keywords:** mitophagy, mitochondrial biogenesis, mitochondrial dynamics, mitochondrial quality control, sepsis, traditional Chinese medicine

## Abstract

Sepsis is a life-threatening syndrome caused by a dysregulated host response to infection and represents a major challenge in critical care medicine due to its high global mortality. Mitochondrial quality control (MQC) denotes the regulatory network essential for maintaining mitochondrial homeostasis, encompassing processes including mitochondrial biogenesis, mitophagy, and mitochondrial dynamics. Impaired MQC leads to the accumulation of damaged mitochondria and constitutes a core pathological mechanism underlying sepsis-induced multiorgan dysfunction. Recent studies have demonstrated that various bioactive components of traditional Chinese medicine (TCM) can facilitate the restoration of the MQC network under septic conditions by modulating distinct signaling pathways, thereby attenuating cytokine storms, mitigating oxidative stress, and preserving organ function. Owing to their multicomponent and multitarget characteristics, TCM interventions offer promising therapeutic potential for sepsis. However, a systematic understanding of how these agents coordinately regulate MQC remains limited. In this review, we aim to elucidate the pharmacological roles and underlying molecular mechanisms of TCM in modulating mitochondrial quality during sepsis, and we also discuss future research directions in this field.

## 1 Introduction

Sepsis is defined as life-threatening organ dysfunction caused by a dysregulated host response to infection (Singer et al., 2016). Its high incidence and mortality make it a major challenge in critical care medicine worldwide, including in China. Data from the Global Burden of Disease study indicate that sepsis continues to impose a substantial burden of illness and death globally (Gray et al., 2025). In addition, multicentre epidemiological surveys conducted in intensive care units (ICUs) across China confirm that sepsis remains a leading cause of mortality among ICU patients (Xie et al., 2020). Despite improvements in diagnostic accuracy, anti-infective therapies, and organ support strategies—along with an increasing number of multicentre randomized controlled trials—a systematic review of sepsis intervention studies from the Sepsis-3 era revealed that most novel therapeutic approaches have failed to significantly reduce overall mortality (Nemet et al., 2025). Sepsis often progresses rapidly and is strongly associated with secondary multiple organ dysfunction syndrome (MODS). Currently, effective targeted interventions remain limited, contributing to persistently high mortality rates and underscoring the urgent need for better therapeutic strategies.

Mitochondria, often described as cellular “powerhouses,” play a central role in sepsis-induced organ dysfunction through disruption of mitochondrial quality control (MQC). On the one hand, dysfunctional mitochondria fail to meet the heightened energy demands of activated immune cells and highly metabolic organs, directly precipitating a cellular “energy crisis” and metabolic collapse that ultimately contributes to multiple organ dysfunction (Lira Chavez et al., 2023). On the other hand, damaged mitochondria release mitochondrial damage-associated molecular patterns (mtDAMPs), such as mitochondrial DNA (mtDNA) and reactive oxygen species (mtROS). These molecules directly activate key intracellular inflammatory signaling pathways, including the NLRP3 inflammasome and the cGAS-STING axis, thereby triggering and amplifying a cytokine storm (Hu et al., 2024). Research has indicated that a self-reinforcing vicious cycle develops between impaired mitochondrial quality control and excessive NLRP3 inflammasome activation. This cycle serves as a critical pathological foundation for the progression of sepsis towards a hyperinflammatory state and the loss of cellular homeostasis (Liao et al., 2026). Consequently, therapeutic strategies aimed at restoring mitochondrial quality control homeostasis are considered promising approaches for improving outcomes in sepsis.

Research on interventions targeting mitochondrial dysfunction in sepsis has emerged as a prominent focus in critical care medicine. To date, most studies have concentrated on single pathways or molecular targets—for example, the use of specific inhibitors or agonists to regulate PINK1/Parkin-mediated mitophagy or Dynamin-related protein 1 (DRP1) -mediated mitochondrial fission (Yaribeygi et al., 2023). Although such “single-target” strategies are valuable for elucidating specific molecular mechanisms, the MQC network in sepsis is characterized by multidimensional and stage-specific dynamic dysregulation. As a result, single interventions are often insufficient to address the complex pathophysiology of the condition. Traditional Chinese medicine (TCM), whose components are primarily derived from natural herbs and animal sources, offers a multitarget therapeutic approach. Accumulating evidence suggests that various bioactive TCM compounds and formulations can differentially modulate mitophagy, mitochondrial dynamics, and biogenesis, thereby ameliorating sepsis-induced damage to multiple organs, including the heart, lungs, kidneys, and intestines (Wang J. et al., 2025; Wang Z. et al., 2025; Song et al., 2025; Wei et al., 2023).

Significantly, while the paradigm of TCM-mediated MQC regulation has been well-established in chronic conditions such as cardiovascular diseases (Pan et al., 2025), and the overall therapeutic value of TCM against sepsis-induced organ dysfunction has been widely recognized (Song et al., 2023), a critical gap remains. Current literature lacks a systematic integration that maps how these multi-target TCM interventions interact with the intricate MQC network specifically during acute septic challenges. To address this, this review systematically summarizes and discusses the pharmacological effects and mechanisms through which TCM regulates mitochondrial quality control-related molecules in the context of sepsis. In addition, we analyze key herbal components involved in these therapeutic effects and propose future research directions in this emerging field.

### 1.1 Search strategy

To ensure a comprehensive and rigorous literature collection, we systematically searched the PubMed database for peer-reviewed articles published in English up to May 2026. A progressive three-tiered search strategy was designed to capture the intersection of sepsis, MQC and TCM. The specific search strings were as follows:

Phase 1 (“sepsis”[Mesh] OR “septic shock” OR “endotoxemia” OR “multiple organ dysfunction syndrome”) AND (“mitochondrial quality control” OR “mitophagy” OR “mitochondrial dynamics” OR “mitochondrial fission” OR “mitochondrial fusion” OR “mitochondrial biogenesis”).

Phase 2 (“sepsis” OR “septic shock”) AND (“mitochondrial quality control” OR “mitophagy” OR “mitochondrial dynamics” OR “mitochondrial biogenesis”) AND (“traditional Chinese medicine”[Mesh] OR “herbal medicine” OR “extracts”).

Phase 3 (“traditional Chinese medicine” OR “herbal medicine” OR “extracts”) AND (“PINK1” OR “Parkin” OR “BNIP3” OR “NIX” OR “FUNDC1” OR “BCL2L13” OR “FKBP8” OR “MFN1” OR “MFN2” OR “OPA1” OR “DRP1” OR “FIS1” OR “PGC-1α” OR “TFAM” OR “SIRT1” OR “SIRT3” OR “STAT3”).

### 1.2 Inclusion and exclusion criteria

Studies were included if they investigated the effects of TCM, herbal compounds, or natural products on MQC-related processes, including mitophagy, mitochondrial dynamics, or mitochondrial biogenesis, in experimental models.

Eligible studies included sepsis models as well as other disease or stress models involving inflammation, oxidative stress, or mitochondrial dysfunction, when mechanistic insights into MQC regulation were provided.

Studies without relevance to MQC mechanisms or lacking experimental evidence were excluded.

## 2 Regulation of mitochondrial quality control

During the early phase of sepsis, the body enters a state of hypermetabolism that is often accompanied by secondary inhibition of mitochondrial respiratory chain function (Cheng et al., 2016). Clinical studies have shown that the activity of mitochondrial complex I is significantly reduced in patients with septic shock, and the extent of this reduction is positively correlated with 28-day mortality (Brealey et al., 2002). Such impairment decreases ATP synthesis, thereby precipitating a crisis in tissue energy metabolism. Moreover, mitochondrial dysfunction increases electron leakage from the electron transport chain (Rosa et al., 2023; McLaughlin et al., 2018). When the defensive capacity of the endogenous antioxidant system (e.g., superoxide dismutase, glutathione peroxidase) becomes depleted or relatively insufficient at this stage, the balance between reactive oxygen species generation and clearance is disrupted, ultimately inducing oxidative stress (DiGiovanni et al., 2025).

### 2.1 Abnormalities in mitophagy

Mitophagy is a highly selective autophagic process through which dysfunctional or structurally abnormal mitochondria are specifically identified, engulfed, and degraded through autophagy-related mechanisms. This process is essential for maintaining mitochondrial quality, energy metabolic homeostasis, and cell survival (Onishi et al., 2021). The concept was formally introduced by Lemasters in 2005 to distinguish this process from general autophagy, thereby underscoring its targeted nature and biological specificity toward mitochondria (Lemasters et al., 2005). Key inducers of mitophagy include oxidative stress (e.g., the accumulation of ROS/RNS (Guo et al., 2025; Feng et al., 2017), inflammatory cytokines (e.g., IL-6) (Tyrrell and Goldstein, 2021), mitochondrial DNA mutations (Frison et al., 2025), and nutrient deprivation (Zhang Y. et al., 2026).

#### 2.1.1 Ubiquitin-dependent mitophagy

The PINK1–Parkin pathway represents the canonical mechanism underlying ubiquitin-dependent mitophagy. Under physiological conditions, the PINK1 translocase complex (TOM/TIM23) transports PINK1 into the inner mitochondrial membrane, where it is cleaved by the protease PARL and subsequently released into the cytosol for proteasomal degradation. This process maintains low basal levels of PINK1 (Neupert and Herrmann, 2007; Callegari et al., 2025; Jin et al., 2010; Yamano and Youle, 2013). Following mitochondrial damage, the loss of membrane potential (ΔΨm) blocks PINK1 import, leading to its stabilization and accumulation on the outer mitochondrial membrane (OMM). PINK1 forms homodimers and undergoes autophosphorylation (e.g., at Thr202/Ser228), thereby becoming an active kinase (Okatsu et al., 2012; Gan et al., 2022). Activated PINK1 first phosphorylates ubiquitin moieties on the OMM, generating pSer65-Ub, which then recruits cytosolic Parkin with high affinity (Koyano et al., 2014; Kazlauskaite et al., 2014; Lazarou et al., 2015). Concurrently, PINK1 phosphorylates the ubiquitin-like (UBL) domain of Parkin (at Ser65). Together with pSer65-Ub, this phosphorylation relieves the autoinhibitory conformation of and fully activates its E3 ubiquitin ligase activity (Condos et al., 2018; Kumar et al., 2015; Sauvé et al., 2018; Panicker et al., 2017; Marchesan et al., 2024). Activated Parkin then catalyses the formation of K63-linked polyubiquitin chains on OMM proteins such as VDAC1 and mitofusin 1/2 (MFN1/2) (Koyano et al., 2014; Wendrich et al., 2025). These ubiquitin chains are specifically recognized by autophagy receptor proteins (e.g., OPTN and NDP52), which bind to LC3/GABARAP proteins via their LC3-interacting region (LIR) motifs, thereby leading to the engulfment of damaged mitochondria by autophagosomes and eventual degradation upon lysosomal fusion (Lazarou et al., 2015; Moore and Holzbaur, 2016; Padman et al., 2019; Ashrafi and Schwarz, 2013; Evans and Holzbaur, 2020).

Signal amplification in this pathway is achieved through a dual positive feedback mechanism. At the post-translational level, Parkin ubiquitinates OMM proteins, generating additional substrates for PINK1 (Zhuang et al., 2016; Wauer et al., 2015a). At the transcriptional level, PINK1 phosphorylates SMAD3 (at Ser423/425), promoting its nuclear translocation and binding to ARE elements, which upregulates PINK1 expression (Tang M. et al., 2025). Negative regulation is mediated by the deubiquitinating enzyme USP30, which reverses ubiquitination but is competitively inhibited by the “phospho-cap” structure of pSer65-Ub (Buus et al., 2009; Kazi et al., 2025; Wauer et al., 2015b). Additionally, PTEN-Long can directly dephosphorylate pSer65-Ub (Wang et al., 2018). Collectively, these findings demonstrate that the PINK1–Parkin pathway achieves precise spatiotemporal control of mitophagy through a dynamic balance among phosphorylation, deubiquitination, and dephosphorylation.

#### 2.1.2 Non-ubiquitin-dependent mitophagy

In non-ubiquitin-dependent mitophagy pathways, OMM receptors play a key role. Receptors such as BNIP3, NIX, FUNDC1, BCL2L13, and FKBP8 directly bind to LC3/GABARAP family proteins on the autophagosome membrane via their conserved LIR, thereby anchoring damaged mitochondria to the forming autophagosome and mediating their clearance (Wang et al., 2023; Schweers et al., 2007).

BNIP3 and NIX are among the best-characterized mitophagy receptors and are strongly induced under hypoxic stress through HIF-1α-dependent transcriptional activation (Yasuda et al., 1999; Zhang and Ney, 2009; Bacon et al., 2007). Both proteins localize to the OMM and promote mitophagy through direct interaction with LC3/GABARAP proteins (Roperto et al., 2019; Novak et al., 2010). FUNDC1 is another hypoxia-responsive mitophagy receptor whose activity is regulated through phosphorylation-dependent modulation of LC3 binding (Lv et al., 2017; Kuang et al., 2016; Liu et al., 2012). Under mitochondrial stress conditions, dephosphorylation of FUNDC1 promotes mitophagy activation (Qi Y. et al., 2025; Ma et al., 2020). In addition, BCL2L13 and FKBP8 have also been reported to mediate Parkin-independent mitophagy pathways (Otsu et al., 2015; Murakawa et al., 2015; Bhujabal et al., 2017; Aguilera et al., 2022).

Mitophagy plays a complex and context-dependent role during sepsis progression. In the early stages of sepsis, moderate activation of mitophagy contributes to the removal of dysfunctional mitochondria and limits oxidative stress-induced injury (Liu et al., 2019). Both hypoxic microenvironments caused by microcirculatory dysfunction and excessive oxygen exposure during intensive care may further disrupt mitochondrial homeostasis and influence mitophagy activity during sepsis progression (Paunikar and Chakole, 2024). However, excessive or sustained mitophagy may lead to depletion of functional mitochondria, thereby aggravating cellular energy failure and organ dysfunction (Lelubre and Vincent, 2018; Iba et al., 2024; Zhu et al., 2021). Conversely, impaired mitophagy can also exacerbate inflammatory injury through the accumulation of damaged mitochondria.

Previous studies have shown that lipopolysaccharide (LPS) and interferon-γ (IFN-γ) can suppress PINK1-dependent mitophagy through STAT1-mediated activation of inflammatory caspases (Patoli et al., 2020). Defective mitochondrial clearance subsequently promotes the accumulation of mtROS, cytochrome c, and mtDNA, which contribute to activation of the NLRP3 inflammasome and amplification of inflammatory signaling (Han et al., 2021; Li J. et al., 2023). Furthermore, mtDNA released from damaged mitochondria may activate both NLRP3 and TLR9 signaling pathways, thereby further aggravating systemic inflammatory responses and organ injury during sepsis (Paik et al., 2025).

### 2.2 Dysregulation of mitochondrial dynamics

The morphology, number, and spatial distribution of mitochondria are constantly reshaped through a highly coordinated cycle of fusion and fission—a process termed mitochondrial dynamics. This enables them to form a network that can rapidly respond to cellular energy demands, playing a central role in maintaining metabolic homeostasis and enhancing their adaptive capacity to stress (Tilokani et al., 2018; Chen et al., 2023). At the mechanistic level, this dynamic process is governed by a multilayered regulatory network in which fusion, fission, axonal transport, and mitophagy are tightly coordinated. Through the integrated action of upstream signals, this network helps maintain mitochondrial integrity and cellular homeostasis (Chen et al., 2023).

#### 2.2.1 Mitochondrial fusion

Mitochondrial fusion involves the sequential merging of the outer and inner mitochondrial membranes, ultimately combining two or more mitochondria into an elongated structure. This process not only increases the effective area of the inner membrane and enhances oxidative phosphorylation efficiency but also facilitates the exchange and complementation of matrix contents, membrane lipids, and mtDNA between mitochondria. These actions help maintain functional stability and preserve genome integrity (Griffin et al., 2006; Dorn, 2020; Tábara et al., 2024).

##### 2.2.1.1 Outer mitochondrial membrane fusion

Fusion of the OMM is primarily mediated by the dynamin-like GTPases MFN1 and MFN2 (Huang S. J. et al., 2025; Zacharioudakis et al., 2022). Located on the OMM, MFN1 and MFN2 promote mitochondrial tethering and membrane fusion through GTP hydrolysis-dependent conformational changes (Sloat et al., 2019). Although these proteins share considerable structural similarity, MFN2 additionally participates in mitochondria-associated endoplasmic reticulum membrane (MAM) formation, thereby contributing to calcium signaling, lipid transfer, and mitochondrial metabolic homeostasis (Naón et al., 2023), (Chen et al., 2003). In addition to protein-mediated regulation, lipid signaling also participates in the control of mitochondrial dynamics. MitoPLD, an OMM-localized phospholipase, generates phosphatidic acid (PA), which contributes to mitochondrial membrane remodeling and coordinates the balance between mitochondrial fusion and fission. Through this mechanism, MitoPLD integrates lipid signaling into the broader MQC regulatory network (Baba et al., 2014; Adachi et al., 2016; Zhang et al., 2016).

##### 2.2.1.2 Inner mitochondrial membrane fusion

Fusion of the inner mitochondrial membrane mainly depends on optic atrophy 1 (OPA1), a dynamin-related GTPase localized to the inner mitochondrial membrane (Liu and Chan, 2017; Song et al., 2007; Noh et al., 2020). OPA1 is essential for maintaining cristae architecture, mitochondrial membrane integrity, and respiratory chain stability (Lee and Yoon, 2018; Wang et al., 2021; Ge et al., 2020; Anand et al., 2014). Cardiolipin, a phospholipid enriched in the inner mitochondrial membrane, also plays an important role in mitochondrial signaling and quality control (Kameoka et al., 2018; Thatavarthy et al., 2025). Upon mitochondrial damage, externalization of cardiolipin from the inner to the outer mitochondrial membrane may function as an “eat-me” signal that facilitates mitophagy and the clearance of dysfunctional mitochondria (Chu et al., 2013; Zhang et al., 2025a). In addition to its role in membrane fusion, OPA1 contributes to the preservation of mitochondrial membrane potential and oxidative phosphorylation efficiency (Del Dotto et al., 2018). Under conditions of mitochondrial stress or membrane depolarization, dysregulated OPA1 processing may impair fusion and promote mitochondrial fragmentation (Alavi and Fuhrmann, 2013; Chen et al., 2009).

#### 2.2.2 Mitochondrial fission

Mitochondrial fission is the process through which a mitochondrion divides into daughter mitochondria, thereby facilitating mitochondrial redistribution, turnover, and quality control (Tilokani et al., 2018; Mokhtari et al., 2022; Adebayo et al., 2021). Physiologically, fission enables the segregation of damaged mitochondrial components and promotes their subsequent elimination through mitophagy. Balanced coordination between mitochondrial fusion and fission is therefore critical for maintaining mitochondrial homeostasis and cellular metabolic stability.

DRP1 is the principal mediator of mitochondrial fission (Wang N. et al., 2025; You et al., 2025). Under stress conditions, DRP1 translocates from the cytoplasm to the outer mitochondrial membrane, where it interacts with receptor proteins including mitochondrial fission factor (MFF), mitochondrial dynamics proteins of 49 and 51 kDa (MiD49/51), and fission protein 1 (FIS1) (Liu A. et al., 2024; Losón et al., 2013; Atkins et al., 2016; Otera et al., 2010; Jin et al., 2017). Subsequent oligomerization of DRP1 at mitochondrial constriction sites drives membrane scission and mitochondrial fragmentation (Kage et al., 2022). In addition, mitochondria-associated endoplasmic reticulum contact sites participate in the initiation of mitochondrial constriction and facilitate fission-related membrane remodeling (Korobova et al., 2013).

Mitochondrial dynamics are highly coordinated processes that adapt to cellular demands and stress conditions. Multiple regulatory pathways participate in maintaining the balance between mitochondrial fusion and fission. At the molecular level, post-translational modifications rapidly regulate these processes. For example, CDK1 phosphorylates DRP1 at Ser616 to promote mitochondrial fission (Ta et al., 2007), whereas PKA-mediated phosphorylation of DRP1 at Ser637 inhibits fission (Yu et al., 2019). In contrast, SIRT3 promotes mitochondrial fusion by deacetylating OPA1 (Samant et al., 2014). In addition, energy-sensing pathways such as AMP-activated protein kinase (AMPK)/mTOR and transcriptional regulators including Peroxisome proliferator-activated receptor gamma coactivator-1 alpha (PGC-1α) help coordinate mitochondrial morphology and function according to cellular metabolic needs (Kim et al., 2011; Cao et al., 2025).

Under conditions of systemic inflammation and stress, such as sepsis, the homeostatic balance of mitochondrial dynamics is significantly disrupted (Liu et al., 2019). The core feature is an imbalance of fission and fusion. On the one hand, DRP1 becomes overactivated, with increased translocation of its active form—marked by elevated phosphorylation at Ser616—to mitochondria, leading to excessive fission and fragmentation. On the other hand, the expression of key fusion mediators—MFN1, MFN2, and OPA1—is downregulated, impairing the fusion process (Haileselassie et al., 2019; Xiong et al., 2025). This imbalance directly compromises mitochondrial function, resulting in marked reductions in membrane potential (ΔΨm), ATP production, and excessive ROS production. These functional abnormalities ultimately contribute to dysfunction in key parenchymal cells such as renal tubular epithelial cells, vascular endothelial cells, and cardiomyocytes, thereby exacerbating the pathological progression of multiorgan injury (Brooks et al., 2009; Perry et al., 2018; Zhou and Toan, 2020; Ong et al., 2010).

### 2.3 Impaired mitochondrial biogenesis

Mitochondrial biogenesis is a dynamic process through which cells increase their mitochondrial number and optimize their structure and function in response to physiological or pathological stimuli. This process involves a multilayered regulatory network (Cao et al., 2025; Ye et al., 2025) and requires coordinated expression of both the nuclear and mitochondrial genomes (Scarpulla et al., 2012), along with protein synthesis and import (Shpilka et al., 2021), as well as lipid metabolism (Mesmin, 2016). Dysregulation of mitochondrial biogenesis is a key feature of several pathological conditions, including aging, type 2 diabetes, neurodegenerative diseases, and cardiovascular disorders (Patti et al., 2003; Højlund et al., 2008; Yang et al., 2025; Lee and Wei, 2005). Physiological stimuli such as exercise (Abrego-Guandique et al., 2025), caloric restriction (Civitarese et al., 2007), and cold exposure (Larson et al., 2020) can effectively promote biogenesis, whereas factors such as ageing (Ljubicic and Hood, 2009), nutrient excess (Bramlage et al., 2021), and chronic inflammation (Li et al., 2015) suppress it. Consequently, this process has emerged as a promising therapeutic target.

PGC-1α is widely regarded as the master regulator of mitochondrial biogenesis (Lagouge et al., 2006; Qian et al., 2024; Ventura-Clapier et al., 2008). Acting as a transcriptional coactivator, PGC-1α cooperates with nuclear respiratory factors 1 and 2 (NRF1/2) to promote the expression of mitochondrial transcription factor A (TFAM), a key regulator of mtDNA replication and transcription (Gugneja et al., 1996; Shahannaz et al., 2026; Zhang et al., 2025b). Through this regulatory network, PGC-1α coordinates mitochondrial renewal with cellular energy demands and metabolic adaptation.

The activity of mitochondrial biogenesis is tightly linked to cellular nutrient and energy status. AMPK, an important energy sensor activated during metabolic stress, promotes PGC-1α signaling under conditions of ATP depletion (Chen J. et al., 2025). In parallel, silent information regulator 1 (SIRT1), a NAD +-dependent deacetylase, enhances the transcriptional activity of PGC-1α through deacetylation (Cantó et al., 2009; Rodgers et al., 2005). Together, these pathways integrate metabolic stress signals with mitochondrial adaptive responses and contribute to the maintenance of cellular homeostasis.

Accumulating evidence indicates that mitochondrial biogenesis is profoundly impaired during sepsis (Carré and Singer, 2008; Carchman et al., 2013). Reduced expression of PGC-1α, NRF1, and TFAM has been observed in multiple experimental models of septic injury and is closely associated with defective ATP production, oxidative stress, and mitochondrial dysfunction (Tran et al., 2011; Li H. et al., 2023). Impaired mitochondrial biogenesis further contributes to organ dysfunction in sepsis, particularly in the heart, kidney, and lung (Kuang et al., 2024; Tang R. et al., 2025; Chen W. et al., 2025). Conversely, restoration of mitochondrial biogenesis may facilitate mitochondrial recovery, improve cellular energy metabolism, and attenuate sepsis-induced organ injury (Jin et al., 2020; Wang et al., 2026; Lai et al., 2025). Consequently, therapeutic strategies targeting mitochondrial biogenesis have attracted increasing attention as potential approaches for restoring MQC homeostasis during sepsis.

## 3 Regulation of mitochondrial quality control by traditional Chinese medicine

### 3.1 Regulation of mitophagy

#### 3.1.1 Regulation of the PINK1/Parkin mitophagy pathway

In the pathological progression of sepsis, PINK1/Parkin-mediated mitophagy acts as a double-edged sword. Moderate mitophagy serves as a protective mechanism that clears damaged mitochondria and maintains cellular function. However, either excessive mitophagy or insufficient mitophagy can exacerbate energy failure and promote cell death. A growing body of evidence suggests that TCM interventions do not simply activate or inhibit this pathway. Instead, they act as “homeostatic regulators,” making precise, bidirectional adjustments according to the pathological state of different organs and the stage of injury.

On the one hand, in organs with high energy demands such as the heart, where sepsis frequently induces the accumulation of dysfunctional mitochondria, TCM interventions primarily support protective mitophagy. Kaempferol delivered via a reactive oxygen species-responsive nanosystem (DTM@KA NPs) (Wen et al., 2024), the Po-Ge-Jiu-Xin decoction (PGJXD) (Wang Z. et al., 2025), puerarin (Chang et al., 2022), and the Qiangxin No. 1 formula (QX1) (Huang et al., 2026) act through distinct regulatory mechanisms to converge on the PINK1/Parkin pathway, thereby facilitating the clearance of damaged mitochondria and cellular energy metabolism. DTM@KA NPs activate the TOM20–STAT6–DRP1 axis to promote PINK1/Parkin-dependent mitophagy, whereas QX1 enhances this pathway through CaMKI signaling. PGJXD promotes PINK1 expression and Parkin translocation to mitochondria, thereby facilitating the completion of mitophagic flux. Puerarin has also been reported to activate PINK1/Parkin-dependent mitophagy while simultaneously modulating mitochondrial dynamics through changes in DRP1 and MFN1. These interventions may therefore enhance the removal of damaged mitochondria and alleviate myocardial injury. This protective enhancement is also observed in extra-cardiac tissues; Banxia Xiexin Decoction (BXD) upregulates PINK1/Parkin-dependent mitophagy to restore intestinal barrier function (Dai et al., 2025), while Icaritin promotes mitochondrial clearance to alleviate inflammatory cellular injuries (Luo et al., 2024).

On the other hand, under severe inflammatory stress in organs such as the lungs and kidneys, mitophagy may become overactivated and paradoxically exacerbate cell injury. In these cases, TCM shifts towards inhibiting pathological mitophagy. For instance, Liang-Ge-San (LGS) (Yu et al., 2024) and protopine (PTP) (Xiao et al., 2023) suppress excessive PINK1/Parkin-dependent mitophagy through distinct mechanisms, thereby reducing oxidative stress and inflammatory injury. Similarly, Baoshen Rehabilitation Granules (BSRG) mitigate excessive PINK1/Parkin-mediated mitophagy, reducing apoptosis and inflammation in renal tubular epithelial cells and thereby alleviating sepsis-induced acute kidney injury (Huang R. et al., 2025).

In summary, TCM regulates PINK1/Parkin-dependent mitophagy through multiple components and targets, exerting protective effects against sepsis-related injury in the heart, lungs, intestines, kidneys, and other organs. However, the direction and intensity of this regulation have a double-edged nature: moderate activation clears damaged mitochondria and maintains cellular homeostasis, whereas excessive activation or insufficient mitophagic activity may exacerbate organ injury. Therefore, precise modulation of mitophagy levels according to the specific pathological stage and target organ characteristics represents an important research direction for the prevention and treatment of sepsis with TCM.

#### 3.1.2 Regulation of PINK1/Parkin-independent mitophagy pathways

In the complex pathological setting of sepsis, in which multiple organs have varying needs, the advantage of TCM lies not only in modulating the classic PINK1/Parkin pathway but also in engaging noncanonical receptors and signaling axes—such as BNIP3, FUNDC1, and BCL2L13—to form a multitarget, finely tuned mitophagy regulatory network.

This precise regulation is first reflected in the context-dependent adjustment of alternative mitophagy levels during acute renal injury. Dahuang Chuanxiong Decoction (DCD) has been reported to regulate BNIP3-mediated mitophagy by directly downregulating BNIP3 expression, thereby alleviating excessive mitophagic activation and helping maintain mitochondrial homeostasis under inflammatory stress (Song et al., 2025). Conversely, honokiol (HKL) has been shown to simultaneously downregulate the mitophagy receptors BNIP3, NIX, and FUNDC1, thereby suppressing excessive receptor-mediated mitophagy and reducing cellular apoptosis and downstream fibrotic deposition (Wei et al., 2023). Although this protective brake was originally characterized in chronic renal injury rather than acute inflammation, such homeostatic inhibition encapsulates a generic organelle-stabilizing paradigm, providing a valuable mechanistic framework for predicting how alternative mitophagy fluxes can be manipulated to prevent bioenergetic collapse when hyper-acute septic cascades threaten parenchymal survival.

Beyond epithelial protection, maintaining noncanonical mitochondrial receptor networks represents a crucial defense for rescuing heavily stressed tissues from metabolic collapse. This multi-target synergy is well illustrated by the Danqi Pill (DQP), which activates the FUNDC1–PGAM5–ULK1 axis to promote FUNDC1-mediated mitophagy, thereby optimizing energy metabolism in stressed cardiomyocytes and limiting apoptotic outcomes (Wang X. et al., 2022). Importantly, while these metabolic benefits were documented within a cardiac ischemia/reperfusion environment rather than a septic cascade, this evolutionary conserved engagement of the FUNDC1 network establishes a robust proof-of-concept, offering a promising theoretical blueprint for deploying complex herbal formulas to rescue hyper-inflammatory cardiac depression. Concurrently, Xinyang tablet (XYT) regulates the RIPK3/FUNDC1 axis involved in mitochondrial unfolded protein response and mitophagy (Dong et al., 2024). The herb pair Epimedii Folium and Ligustri Lucidi Fructus(EF & LLF) together target the AMPK/ULK1/BCL2L13 signaling axis to increase mitophagy and reduce oxidative stress, exhibiting greater efficacy than either herb alone (Long et al., 2025).

In addition, an andrographolide derivative (ADA) has been shown to enhance mitochondrial clearance by activating the SIRT3-FOXO3a pathway under endotoxemic stress (Zhou et al., 2024). This pathway increases BNIP3/PINK1 expression and helps restore impaired mitophagy. The Chuanxiong-Danggui herb pair (CDHP) specifically promotes noncanonical mitophagy by upregulating the FUNDC1- PGAM5 axis (Pu et al., 2025). Notably, the marine-derived compound rhopaloic acid A (RA) acts through a distinct modality: it activates the JNK pathway to promote BNIP3/NIX phosphorylation, triggering excessive mitophagy-associated cell death under specific inflammatory conditions (Chen W. F. et al., 2024), thereby offering a new perspective for developing potential intervention strategies that exploit this pathway under specific hyper-inflammatory cascades.

Beyond canonical mitophagy pathways, some TCM agents regulate mitochondrial homeostasis through integrated MQC networks. Astragaloside IV (AS-IV) (Wang J. et al., 2025), for example, protects against septic myocardial injury by activating the DUSP1–PHB2 axis, which coordinates mitophagy and mitochondrial quality control to restore mitochondrial homeostasis.

### 3.2 Regulation of mitochondrial dynamics

An imbalance in mitochondrial dynamics is a key driver of the pathological process of sepsis and occurs in a clearly time-dependent pattern. In the early stage, excessive mitochondrial fission triggers inflammation and oxidative stress. In the later stage, promoting fusion becomes important for driving repair (Liu et al., 2019)^,^ (Wu et al., 2019). In response to this dynamic progression, TCM interventions result in a distinct bidirectional regulatory pattern: inhibition of excessive DRP1-mediated fission in the early stage and promotion of MFN- and OPA1-mediated fusion in the later stage.

#### 3.2.1 Early intervention: inhibiting excessive fission

In the early stage of sepsis, TCM targets the DRP1 network through various mechanisms to suppress excessive fission. Some active components act directly on the function of DRP1. For example, notoginsenoside R1 (NGR1) (Hou et al., 2024a) inhibits the translocation of DRP1 to the mitochondrial outer membrane, whereas tangeretin (TAN) (Liu Y. et al., 2024) upregulates PLK1, activates AMPK, and increases the inhibitory phosphorylation of DRP1 at Ser637, thereby ameliorating excessive mitochondrial fission. Other components regulate upstream or synergistic pathways. For instance, Parthenolide (PTL) (Zhang J. et al., 2025) acts through the BRD4/BCL-xL pathway to simultaneously suppress fission and reduce ROS production. Additionally, curcumin (Hou et al., 2024b) and berberine (BBR) (Qin et al., 2019) exert protective effects through the SIRT1-DRP1/PGC-1α pathway and by inhibiting DRP1 overactivation, respectively. Notably, DTM@KA NPs (Wen et al., 2024) effectively reversed the imbalance in dynamics via the TOM20-STAT6-DRP1 pathway. Moreover, studies on phorbol 12-myristate 13-acetate (PMA) as a PKC-α activator suggest that protein kinase signaling pathways may also represent potential nodes for TCM regulation of mitochondrial dynamics (Li et al., 2018).

#### 3.2.2 Later intervention: promotion of fusion for repair

In the later repair phase, the focus of treatment shifts towards promoting mitochondrial fusion (Lira Chavez et al., 2023). Chimonanthus nitens Oliv. Essential oil (CEO) (Huang S. et al., 2025) suppresses MFN2 overexpression and reduces pathological mitochondria-associated membrane formation, thereby helping restore mitochondrial–ER homeostasis. Other TCM formulas, such as Shen Shuai II Recipe (SSR) (Wang M. et al., 2022), Xin-Ji-Er-Kang (XJEK) (Zhang R. et al., 2026), and Xuanfei Baidu Formula (XBF) (Li Z. et al., 2023), act by activating the PGC-1α-MFN2 axis or upregulating MFN1/2. In doing so, they restore the mitochondrial network while suppressing downstream inflammatory pathways such as mtROS, cGAS-STING, and NLRP3.

Individual active compounds exhibit even more refined regulation. This structural plasticity is remarkably demonstrated by Schisandrin B (Sch B), which bidirectionally modulates both fission and fusion pathways through the site-specific phosphorylation of AKT1 (Xu C. et al., 2025). Although this balancing capacity was originally mapped within chronic cardiac hypertrophy models rather than acute endotoxemia, the underlying molecular mechanism serves as an instructive reference paradigm, offering key insights into how natural agents can be deployed to restore morphological rhythms during acute septic cardiomyopathy. In distinct acute settings, kirenol (Zhang Y. et al., 2025), volvalerenic acid A (VaA) (Ge et al., 2024), and modified Hu-lu-ba-wan (MHLBW) (Gong et al., 2024) all increase OPA1-mediated fusion, albeit through distinct pathways, including those involving CK2/AKT, IDO1, and PKM2-PGC-1α. Additionally, ginsenoside compound K (Huang et al., 2023), Longxuetongluo capsule (LTC) (Yang et al., 2024), and gastrodin (Cheng et al., 2020) help restore the balance of mitochondrial dynamics through various mechanisms, such as blocking MFN2 ubiquitination and degradation, or reducing ROS production. Active components from *Rehmannia glutinosa,* including TOM20/DRP1, AMPK/SIRT1/PGC-1α, and NRF2, exert protective effects through multiple pathways (Zhong et al., 2025). Interestingly, the combination of hypoxic preconditioning and curcumin has a synergistic effect (Wang et al., 2020), suggesting that combined strategies hold significant potential for promoting fusion and repair.

### 3.3 Regulation of mitochondrial biogenesis

During the progression of sepsis, impaired mitochondrial biogenesis, together with imbalances in dynamics and abnormal mitophagy, contributes to cellular energy failure (Chen et al., 2023). In response, TCM has established a multilayered regulatory network—spanning from upstream transcriptional activation to downstream genome maintenance—that systematically restores mitochondrial function.

#### 3.3.1 Transcriptional regulation and energy sensing: the AMPK/SIRT1 and NRF2/PGC-1α hub

A key focus of TCM intervention is the activation of the master transcriptional regulators NRF2 and PGC-1α. On the one hand, various components—such as resveratrol (Kim et al., 2014), phlorotannins (Yang et al., 2016), *Arbutus unedo* aqueous extract (Mariotto et al., 2008), Dahuang Gancao Decoction (DHGCD) (Cui et al., 2026), and Wenqingyin (WQY) (Xie et al., 2023)—activate NRF2 through distinct signaling pathways (e.g., eNOS-NO and HO-1). Collectively, they exert antioxidant, anti-inflammatory, and anti-ferroptotic effects, thereby creating a favorable microenvironment that allows the biogenesis machinery to function. On the other hand, the PGC-1α-centered network represents another key regulatory axis of mitochondrial biogenesis. Curcumin (Hou et al., 2024b) and sodium tanshinone IIA sulfonate (STS) (Zhou et al., 2019) promote mitochondrial biogenesis through the PGC-1α/NRF1/TFAM axis, whereas salvianolic acid C (SAC) (Xu et al., 2026) enhances PGC-1α-related transcriptional activity and increases PCK1 expression. This directly drives the expression of genes involved in mitochondrial replication, effectively restoring respiratory chain capacity and organ energy metabolism.

Notably, several compounds target the same core regulatory machinery under alternative pathophysiological challenges, offering valuable insights into the generic tissue-protective potential of these pathways. For instance, the protective and mitochondrial benefits of carnosol (Li et al., 2024), resveratrol (Kim et al., 2014), secondary metabolites from Rehmannia glutinosa (Zhong et al., 2025), and Arbutus unedo aqueous extract (Mariotto et al., 2008) were primarily validated within models of chronic microvascular remodeling or localized oxidative stress rather than polymicrobial sepsis. Nevertheless, their common capacity to stabilize the SIRT1/NRF2/PGC-1α network under cellular duress provides a useful reference framework, suggesting that these natural agents hold potential for mitigating downstream organ failure if properly integrated into hyper-inflammatory contexts.

Crucially, the upstream AMPK/SIRT1 axis serves as a key energy sensor that links cellular metabolic status to these transcriptional signals. Ginsenoside Rg3 (Xing et al., 2017), Gypenoside XLIX (Gyp-XLIX) (Zhang W. et al., 2025), Zn^2+^-shikonin-PEG nanoparticles (Zn-Shik-PEG NPs) (Guo et al., 2023), and extracellular vesicles derived from Chinese leek (CL-EVs) (Qi W. et al., 2025) all activate the AMPK/SIRT1 axis. This activation directly upregulates PGC-1α activity and, through post-translational modifications such as deacetylation, enhances mitochondrial function, forming a positive feedback loop that integrates sensing and response.

#### 3.3.2 Suppression of inflammation and maintenance of genome stability

Severe systemic inflammation during sepsis acts as a direct biological brake that suppresses mitochondrial biogenesis. TCM formulations effectively release this external inhibition while simultaneously safeguarding downstream mtDNA stability to guarantee functional organelle assembly.

At the inflammatory interface, TCM breaks the negative feedback loops that stifle biogenesis factors. Isoferulic acid (IFA) (Xu T. et al., 2025), extracts from *Physalis angulata* L. (Liu et al., 2025), and Xuebijing injection (XBJ) (Zheng et al., 2020) exert precise inhibitory control over hyperactivated inflammatory nodes, including the NF-κB and JAK2/STAT3 signaling pathways. By dampening these upstream cytokine cascades, these interventions dismantle the molecular barriers that paralyze biogenesis factors such as PGC-1α. This anti-inflammatory relief is complemented by the ethanol extract of Prunella vulgaris var. lilacina, which induces HO-1 expression and restricts high mobility group box 1 (HMGB1) release (Jun et al., 2012), further alleviating the microenvironmental duress that blocks mitochondrial replication.

This comprehensive relief of inflammatory suppression ultimately converges on the protection of TFAM, the key executor of mitochondrial genome replication, transcription, and nucleoid packaging. The Seed oils of *Rosa roxburghii* Tratt (RRSO) (Ni et al., 2022) and songorine (Chen M. et al., 2024) increase TFAM expression through signaling pathways such as PPARα or Wnt/β-catenin pathways. This directly supports mtDNA stability and transcription, which are essential for the reconstruction of mitochondrial function.

Overall, TCM restores mitochondrial biogenesis in sepsis through an integrated regulatory network. This network uses NRF2 and PGC-1α as transcriptional hubs, is fine-tuned by the AMPK/SIRT1 axis according to energy status, relieves external inhibition by suppressing inflammatory signals, and ultimately ensures that TFAM can effectively carry out genome maintenance. Importantly, these interventions do not all directly activate mitochondrial biogenesis; some primarily regulate oxidative stress or inflammatory signaling and thereby create a favorable environment for mitochondrial restoration. This multilayered, multistep integrated regulatory model not only provides a clear framework for understanding the biological mechanisms of TCM in the treatment of sepsis but also lays a solid foundation for the development of precision therapies aimed at restoring mitochondrial homeostasis.

## 4 Perspectives and future

The collapse of the MQC network is considered one of the key pathophysiological foundations underlying the progression of sepsis to MODS. Clinically, patients with sepsis often continue to experience circulatory instability, poor lactate clearance, and increased requirements for organ support even after the infection is controlled. Beyond systemic inflammation, these manifestations reflect a deeper failure of mitochondrial homeostasis, in which impaired repair of damaged organelles leads to progressive bioenergetic collapse. The major MQC processes, including mitophagy, mitochondrial dynamics (fission/fusion), and mitochondrial biogenesis, do not operate independently but instead form an interactive regulatory system whose disruption amplifies cellular dysfunction during sepsis progression.

**FIGURE 1:**
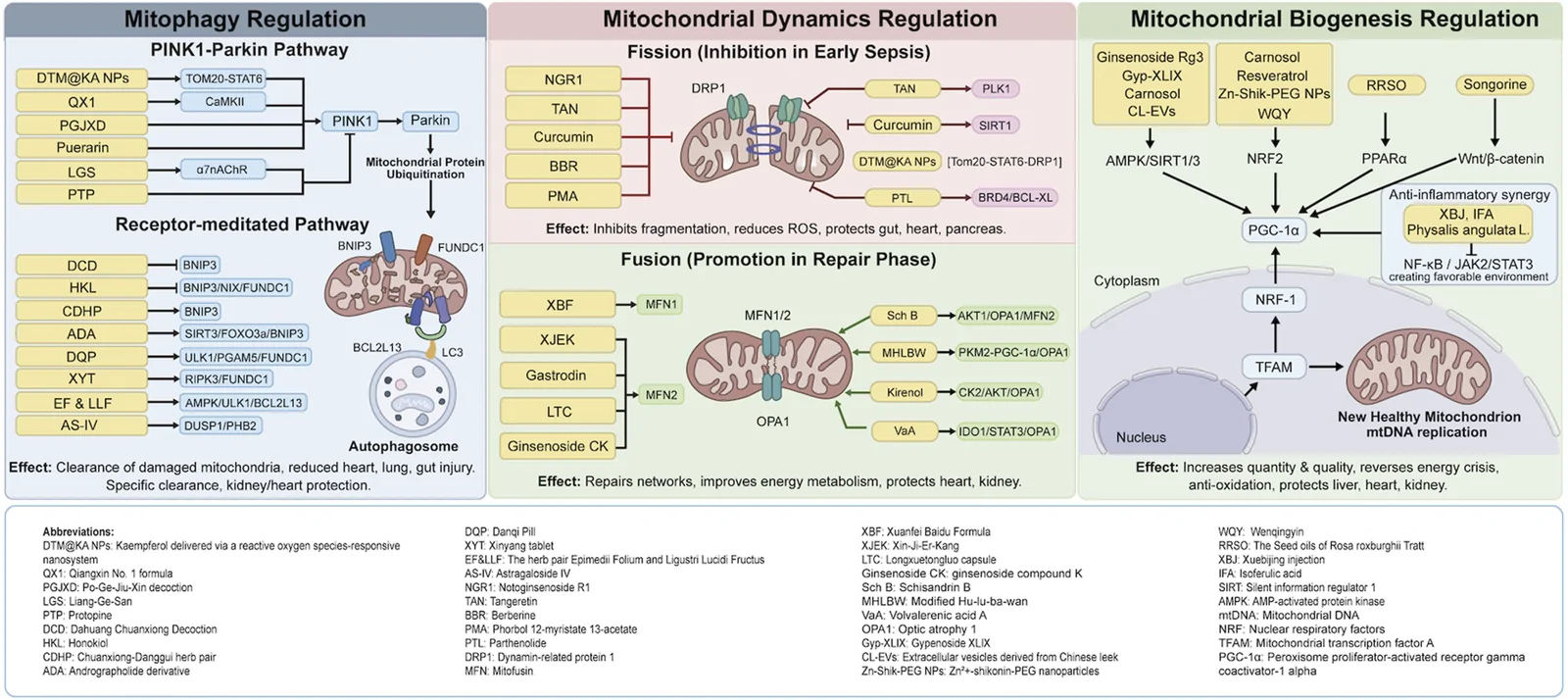
Mechanisms of Traditional Chinese Medicine in Regulating Mitochondrial Quality Control for Sepsis Treatment. This schematic illustrates the key MQC pathways—mitophagy, mitochondrial dynamics, and mitochondrial biogenesis—that are dysregulated during sepsis, and highlights the intervention nodes targeted by various TCM components. TCM exerts multi-target regulatory effects on these pathways, thereby restoring mitochondrial homeostasis, attenuating inflammatory responses and oxidative stress, and ultimately ameliorating sepsis-induced multiple organ dysfunction. Abbreviations: MQC, mitochondrial quality control; TCM, traditional Chinese medicine; ROS, reactive oxygen species; MODS, multiple organ dysfunction syndrome.

Within this framework, TCM appears to exert regulatory effects on MQC through multi-level modulation of mitochondrial function rather than isolated pathway targeting. As shown in Table 1, diverse herbal compounds and formulas simultaneously affect mitophagy, mitochondrial dynamics, oxidative stress, and mitochondrial biogenesis, thereby contributing to partial restoration of mitochondrial stability under septic stress. For example, puerarin has been reported to regulate both PINK1/Parkin-dependent mitophagy and DRP1-mediated mitochondrial fission, while ginsenosides may support mitochondrial biogenesis alongside preservation of mitochondrial dynamics. These observations are consistent with the integrated MQC regulatory network illustrated in Figure 1.

**TABLE 1:** Representative traditional Chinese medicine (TCM) agents targeting mitochondrial quality control in sepsis.

MQC process/Category	TCM intervention/compound	Experimental model	Mitophagy pathway/target	Primary protective effect	Ref. no.
Mitophagy					
Single compounds and isolates	Astragaloside IV	LPS-induced SCM mice & H9C2 cells	Upregulates DUSP1/PHB2 axis; regulates mito-ER cross-talk	Restores mitochondrial homeostasis	Wang J. et al. (2025)
​	Honokiol (HKL)	LPS-induced AKI mice & HK-2 cells	Activates SIRT3; enhances PINK1/Parkin-mediated mitophagy	Accelerates damaged organelle clearance	Wei et al.(2023)
Complex formulas and decoctions	Banxia Xiexin decoction (BXD)	CLP-induced sepsis mice & Caco-2 cells	Activates PINK1/Parkin signaling pathway	Restores intestinal barrier function	Dai et al. (2025)
​	Dahuang Chuanxiong decoction (DCD)	LPS-induced AKI rats & HK-2 cells	Activates noncanonical BNIP3-mediated mitophagy	Promotes mitochondrial clearance	Song et al.(2025)
​	Liang-Ge-San (LGS)	LPS-induced ALI mice & MLE-12 cells	Suppresses hyperactivated PINK1/Parkin pathway	Restrains pathological mitophagy	Yu et al. (2024)
​	Baoshen rehabilitation granules (BSRG)	CLP-induced AKI mice	Inhibits overactivated Parkin-dependent mitophagy	Alleviates tubular cell apoptosis	Huang R. et al. (2025)
Dynamics					
Single compounds and isolates	Notoginsenoside R1 (NGR1)	CLP-induced polymicrobial sepsis mice	Modulates baseline DRP1 expression and mitochondrial localization	Suppresses excessive fission	Hou et al. (2024a)
​	Tangeretin (TAN)	LPS-induced endotoxemia mice & RAW264.7	Regulates PLK1/AMPK/DRP1 signaling network	Inhibits anomalous fission	Liu Y. et al. (2024)
​	Parthenolide (PTL)	LPS-induced inflammatory cell models	Modulates upstream BRD4/BCL-xL pathway	Inhibits DRP1-mediated fission	Hou et al. (2024b)
Complex formulas and decoctions	Xuanfei Baidu formula (XBF)	LPS-induced ALI mice	Activates PGC-1α-MFN2 axis; downregulates NLRP3	Promotes structural fusion	Li Z. et al. (2023)
Biogenesis					
Single compounds and isolates	Sodium tanshinone IIA sulfonate (STS)	LPS-induced endothelial injury cells	Activates eNOS-NO & HO-1 signaling via NRF2	Promotes NRF2 translocation	Zhou et al. (2019)
​	Curcumin	LPS-induced endotoxemic shock rats	Upregulates SIRT1/PGC-1αtranscriptional hub	Resuscitates ATP production	Hou et al. (2024b)
​	Ginsenoside Rg3	LPS-induced endotoxemia mice	Upregulates PGC-1α/NRF-1/TFAM signaling cascade	Drives mtDNA replication	Xing et al. (2017)
​	Songorine	LPS-induced cellular stress models	Enhances TFAM expression via PPARα or Wnt	Safeguards mtDNA stability	Chen M. et al. (2024)
Complex formulas and decoctions	Xuebijing injection (XBJ)	CLP-induced systemic sepsis rats	Inhibits hyperactivated NF-κB & JAK2/STAT3 nodes	Releases inflammatory blocks	Zheng et al. (2020)

**FIGURE 2:**
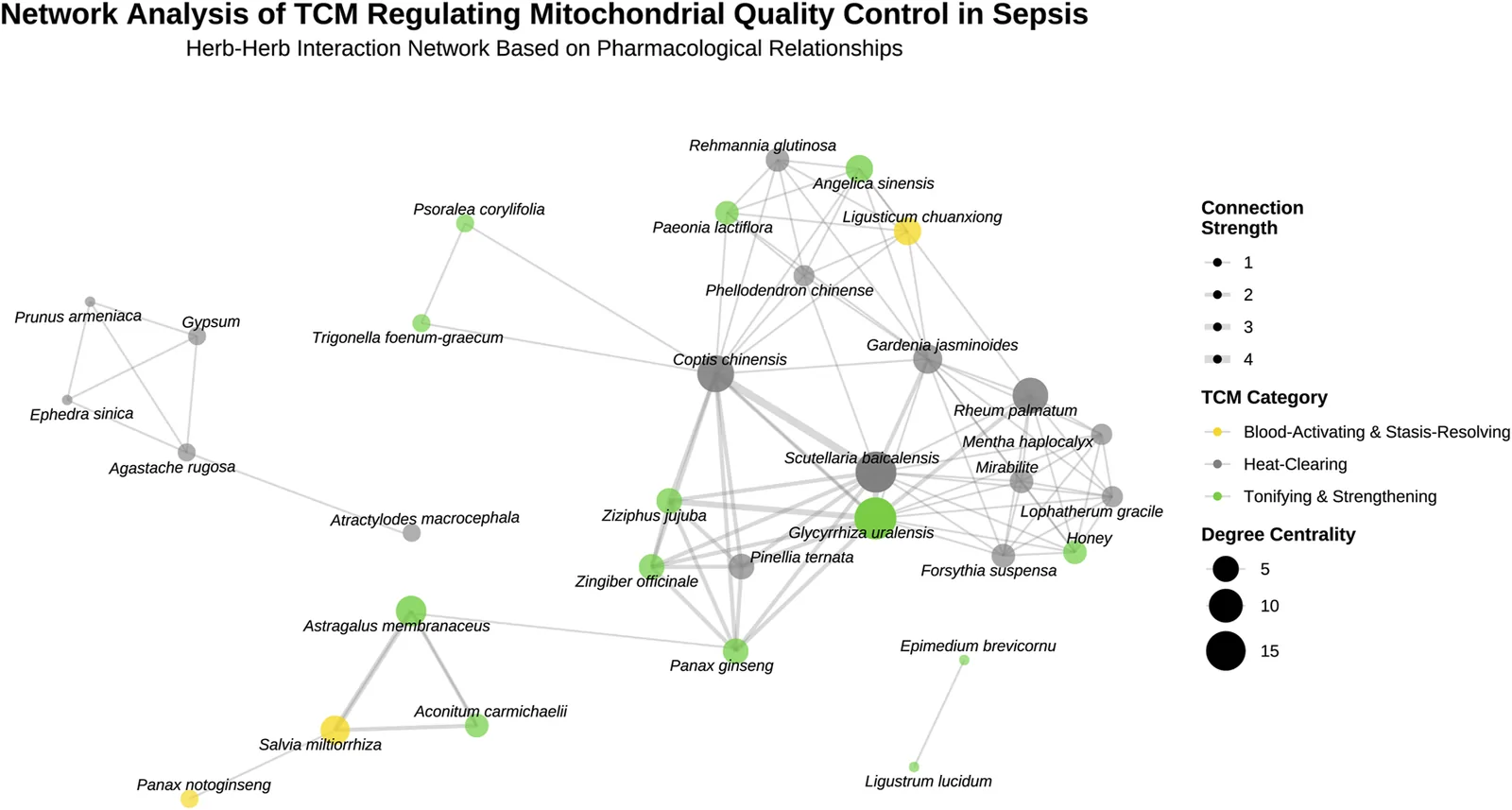
Regulatory Network of Single Medicinal Agents Involved in Mitochondrial Quality Control in Sepsis. This network was constructed based on the relative frequency of research citations. Node size reflects degree centrality, with larger nodes indicating greater connectivity within the network. Connections between nodes represent co-occurrence or association of the respective agents within compound formulas, with line thickness indicating connection strength. Node colors represent different TCM categories. Abbreviations: TCM, traditional Chinese medicine.

In clinical application, TCM interventions are predominantly used in multi-herb combinations rather than as single agents. Such formulations allow functional complementarity across different biological processes, including inflammatory modulation, redox balance, and mitochondrial metabolism. As reflected in Figure 2, single medicinal agents are frequently connected through shared inclusion in classical or experimental formulas, suggesting that their therapeutic relevance is partly defined by combinatorial usage patterns rather than isolated pharmacological activity. However, current evidence remains insufficient to define clear rules governing herb–herb interactions, contraindications, or long-term safety profiles in septic populations.

Temporal and organ-specific variability further complicates MQC regulation during sepsis. Early disease stages are often characterized by excessive mitochondrial fragmentation and uncontrolled mitophagy, whereas later stages shift toward energy depletion and impaired mitochondrial renewal. Accordingly, the functional direction of MQC modulation may differ across disease phases. Moreover, organ-specific metabolic demands influence pathway preference: cardiomyocytes tend to prioritize PINK1/Parkin-associated mitochondrial quality control, whereas renal tissue shows greater involvement of receptor-mediated mitophagy pathways such as BNIP3 and FUNDC1. These differences suggest that MQC regulation is not uniform but context-dependent across both time and tissue.

Despite extensive experimental evidence, clinical translation of MQC-targeted TCM strategies remains limited. Current findings are largely derived from *in vitro* systems and animal models, with a marked lack of well-designed clinical studies assessing mitochondrial endpoints in septic patients. In addition, heterogeneity in experimental design, intervention timing, and outcome measures limits cross-study comparability. The limited availability of pharmacokinetic, dose–response, and toxicity data further complicates the determination of clinically relevant exposure ranges and therapeutic windows. Most importantly, the molecular interactions between active compounds, mitochondrial pathways, and organ-specific responses remain insufficiently defined, hindering precise therapeutic translation.

Future research should therefore shift from descriptive observations toward mechanistic and translational precision. Integrative approaches combining multi-omics profiling, network pharmacology, and single-cell resolution analyzes may help clarify dynamic MQC regulation during sepsis progression. Standardized preparation, pharmacokinetic characterization, dose–response studies, and systematic safety evaluation will also be essential for defining clinically relevant therapeutic windows. Clinically, mitochondrial function–related biomarkers may facilitate patient stratification and therapeutic monitoring. Furthermore, emerging delivery technologies, including nanocarriers and organ-targeted systems, may enhance the specificity and efficacy of bioactive TCM components. Collectively, these advances may help bridge the current gap between experimental findings and clinically actionable mitochondrial-targeted therapies in sepsis.

## References

[B1] Abrego-Guandique D. M. Aguilera Rojas N. M. Chiari A. Luciani F. Cione E. Cannataro R. (2025). The impact of exercise on mitochondrial biogenesis in skeletal muscle: a systematic review and meta-analysis of randomized trials. Biomol. Concepts 16 (1), 20250055. 10.1515/bmc-2025-0055 40459444

[B2] Adachi Y. Itoh K. Yamada T. Cerveny K. L. Suzuki T. L. Macdonald P. (2016). Coincident phosphatidic acid interaction restrains DRP1 in mitochondrial division. Mol. Cell 63 (6), 1034–1043. 10.1016/j.molcel.2016.08.013 27635761 PMC5028122

[B3] Adebayo M. Singh S. Singh A. P. Dasgupta S. (2021). Mitochondrial fusion and fission: the fine-tune balance for cellular homeostasis. FASEB J. 35 (6), e21620. 10.1096/fj.202100067R PMC8415099 34048084

[B4] Aguilera M. O. Robledo E. Melani M. Wappner P. Colombo M. I. (2022). FKBP8 is a novel molecule that participates in the regulation of the autophagic pathway. Biochimica Biophysica Acta (BBA) - Mol. Cell Res. 1869 (5), 119212. 10.1016/j.bbamcr.2022.119212 35090967

[B5] Alavi M. V. Fuhrmann N. (2013). Dominant optic atrophy, OPA1, and mitochondrial quality control: understanding mitochondrial network dynamics. Mol. Neurodegener. 8, 32. 10.1186/1750-1326-8-32 24067127 PMC3856479

[B6] Anand R. Wai T. Baker M. J. Kladt N. Schauss A. C. Rugarli E. (2014). The i-AAA protease YME1L and OMA1 cleave OPA1 to balance mitochondrial fusion and fission. J. Cell Biol. 204 (6), 919–929. 10.1083/jcb.201308006 24616225 PMC3998800

[B7] Ashrafi G. Schwarz T. L. (2013). The pathways of mitophagy for quality control and clearance of mitochondria. Cell Death Differ. 20 (1), 31–42. 10.1038/cdd.2012.81 22743996 PMC3524633

[B8] Atkins K. Dasgupta A. Chen K. H. Mewburn J. Archer S. L. (2016). The role of DRP1 adaptor proteins MiD49 and MiD51 in mitochondrial fission: implications for human disease. Clin. Sci. (Lond). 130 (21), 1861–1874. 10.1042/CS20160030 27660309

[B9] Baba T. Kashiwagi Y. Arimitsu N. Kogure T. Edo A. Maruyama T. (2014). Phosphatidic acid (PA)-preferring phospholipase A1 regulates mitochondrial dynamics. J. Biol. Chem. 289 (16), 11497–11511. 10.1074/jbc.M113.531921 24599962 PMC4036285

[B10] Bacon A. L. Fox S. Turley H. Harris A. L. (2007). Selective silencing of the hypoxia-inducible factor 1 target gene BNIP3 by histone deacetylation and methylation in colorectal cancer. Oncogene 26 (1), 132–141. 10.1038/sj.onc.1209761 16799636

[B11] Bhujabal Z. Birgisdottir Å. B. Sjøttem E. Brenne H. B. Øvervatn A. Habisov S. (2017). FKBP8 recruits LC3A to mediate parkin‐independent mitophagy. EMBO Rep. 18 (6), 947–961. 10.15252/embr.201643147 28381481 PMC5452039

[B12] Bramlage K. S. Bhattacharjee J. Kirby M. Myronovych A. Gupta R. Gonzalez R. M. S. (2021). A diet high in fat and fructose induces early hepatic mitochondrial aging. J. Pediatr. Gastroenterol. Nutr. 73 (1), 99–102. 10.1097/MPG.0000000000003068 PMC8549102 34135298

[B13] Brealey D. Brand M. Hargreaves I. Heales S. Land J. Smolenski R. (2002). Association between mitochondrial dysfunction and severity and outcome of septic shock. Lancet 360 (9328), 219–223. 10.1016/S0140-6736(02)09459-X 12133657

[B14] Brooks C. Wei Q. Cho S. G. Dong Z. (2009). Regulation of mitochondrial dynamics in acute kidney injury in cell culture and rodent models. J. Clin. Invest 119 (5), 1275–1285. 10.1172/JCI37829 19349686 PMC2673870

[B15] Buus R. Faronato M. Hammond D. E. Urbé S. Clague M. J. (2009). Deubiquitinase activities required for hepatocyte growth factor-induced scattering of epithelial cells. Curr. Biol. 19 (17), 1463–1466. 10.1016/j.cub.2009.07.040 19699092 PMC2764384

[B16] Callegari S. Kirk N. S. Gan Z. Y. Dite T. Cobbold S. A. Leis A. (2025). Structure of human PINK1 at a mitochondrial TOM-VDAC array. Science 388 (6744), 303–310. 10.1126/science.adu6445 40080546

[B17] Cantó C. Gerhart-Hines Z. Feige J. N. Lagouge M. Noriega L. Milne J. C. (2009). AMPK regulates energy expenditure by modulating NAD+ metabolism and SIRT1 activity. Nature 458 (7241), 1056–1060. 10.1038/nature07813 19262508 PMC3616311

[B18] Cao L. Li Y. Smirnov A. Voshtani R. Wang T. Shao C. (2025). PGC-1α: key regulator of mitochondrial biogenesis and cellular differentiation in metabolic and regenerative tissues. Cell Biosci. 16 (1), 9. 10.1186/s13578-025-01519-2 41456074 PMC12853593

[B19] Carchman E. H. Whelan S. Loughran P. Mollen K. Stratamirovic S. Shiva S. (2013). Experimental sepsis-induced mitochondrial biogenesis is dependent on autophagy, TLR4, and TLR9 signaling in liver. FASEB J. 27 (12), 4703–4711. 10.1096/fj.13-229476 23982147 PMC3834775

[B20] Carré J. E. Singer M. (2008). Cellular energetic metabolism in sepsis: the need for a systems approach. Biochimica Biophysica Acta (BBA) - Bioenergetics. 1777 (7), 763–771. 10.1016/j.bbabio.2008.04.024 18482575

[B21] Chang X. He Y. Wang L. Luo C. Liu Y. Li R. (2022). Puerarin alleviates LPS-induced H9C2 cell injury by inducing mitochondrial autophagy. J. Cardiovasc Pharmacol. 80 (4), 600–608. 10.1097/FJC.0000000000001315 35881898

[B22] Chen H. Detmer S. A. Ewald A. J. Griffin E. E. Fraser S. E. Chan D. C. (2003). Mitofusins MFN1 and MFN2 coordinately regulate mitochondrial fusion and are essential for embryonic development. J. Cell Biol. 160 (2), 189–200. 10.1083/jcb.200211046 12527753 PMC2172648

[B23] Chen L. Gong Q. Stice J. P. Knowlton A. A. (2009). Mitochondrial OPA1, apoptosis, and heart failure. Cardiovasc Res. 84 (1), 91–99. 10.1093/cvr/cvp181 19493956 PMC2741347

[B24] Chen W. Zhao H. Li Y. (2023). Mitochondrial dynamics in health and disease: mechanisms and potential targets. Signal Transduct. Target Ther. 8, 333. 10.1038/s41392-023-01547-9 37669960 PMC10480456

[B25] Chen W. F. Tsai S. C. Zhang Y. H. Chang H. M. Wu W. J. Su J. H. (2024). Rhopaloic acid A triggers mitochondria damage-induced apoptosis in oral cancer by JNK/BNIP3/Nix-mediated mitophagy. Phytomedicine 132, 155855. 10.1016/j.phymed.2024.155855 39043083

[B26] Chen M. Huang S. Weng S. Weng J. Guo R. Shi B. (2024). Songorine ameliorates LPS-Induced sepsis cardiomyopathy by Wnt/β-catenin signaling pathway-mediated mitochondrial biosynthesis. Naunyn Schmiedeb. Arch. Pharmacol. 397 (7), 4713–4725. 10.1007/s00210-023-02897-5 38133657

[B27] Chen J. Liu B. Yao X. Yang X. Sun J. Yi J. (2025). AMPK/SIRT1/PGC‐1α signaling pathway: molecular mechanisms and targeted strategies from energy homeostasis regulation to disease therapy. CNS Neurosci. Ther. 31 (11), e70657. 10.1111/cns.70657 41268687 PMC12635776

[B28] Chen W. Zhao Z. Geng Z. Zhang H. Fu X. (2025). Advances in mitochondria–nucleus crosstalk in septic cardiomyopathy. Cell Biol. Toxicol. 41 (1), 136. 10.1007/s10565-025-10090-y PMC12500838 41051583

[B29] Cheng S. C. Scicluna B. P. Arts R. J. W. Gresnigt M. S. Lachmandas E. Giamarellos-Bourboulis E. J. (2016). Broad defects in the energy metabolism of leukocytes underlie immunoparalysis in sepsis. Nat. Immunol. 17 (4), 406–413. 10.1038/ni.3398 26950237

[B30] Chu C. T. Ji J. Dagda R. K. Jiang J. F. Tyurina Y. Y. Kapralov A. A. (2013). Cardiolipin externalization to the outer mitochondrial membrane acts as an elimination signal for mitophagy in neuronal cells. Nat. Cell Biol. 15 (10), 1197–1205. 10.1038/ncb2837 24036476 PMC3806088

[B31] Civitarese A. E. Carling S. Heilbronn L. K. Hulver M. H. Ukropcova B. Deutsch W. A. (2007). Calorie restriction increases muscle mitochondrial biogenesis in healthy humans. PLoS Med. 4 (3), e76. 10.1371/journal.pmed.0040076 17341128 PMC1808482

[B32] Condos T. E. Dunkerley K. M. Freeman E. A. Barber K. R. Aguirre J. D. Chaugule V. K. (2018). Synergistic recruitment of UbcH7∼Ub and phosphorylated Ubl domain triggers parkin activation. EMBO J. 37 (23), e100014. 10.15252/embj.2018100014 30446597 PMC6276879

[B33] Cui M. An Y. Zhao J. Hui M. Xiong Z. Guan B. (2026). Dahuang Gancao decoction suppresses ferroptosis via SIRT3-mediated NRF2 deacetylation in acute kidney injury. Phytomedicine 150, 157724. 10.1016/j.phymed.2025.157724 41447840

[B34] Dai W. Zhou L. Hao H. Wang D. Zhang F. Wang P. (2025). Therapeutic effects and molecular mechanism of Banxia Xiexin decoction on intestinal mucosal barrier function in sepsis. Comb. Chem. High. Throughput Screen 29, 1261–1282. 10.2174/0113862073349597250410111941 40304339

[B35] Del Dotto V. Fogazza M. Lenaers G. Rugolo M. Carelli V. Zanna C. (2018). OPA1: how much do we know to approach therapy? Pharmacol. Res. 131, 199–210. 10.1016/j.phrs.2018.02.018 29454676

[B36] DiGiovanni L. F. Khroud P. K. Carmichael R. E. Schrader T. A. Gill S. K. Germain K. (2025). ROS transfer at peroxisome-mitochondria contact regulates mitochondrial redox. Science 389 (6756), 157–162. 10.1126/science.adn2804 40638754

[B37] Dong X. Zhuang H. Wen R. Huang Y. Liang L. B. Li H. (2024). Xinyang tablet alleviated cardiac dysfunction in a cardiac pressure overload model by regulating the receptor-interacting serum/three-protein kinase 3/FUN14 domain containing 1-mediated mitochondrial unfolded protein response and mitophagy. J. Ethnopharmacol. 330, 118152. 10.1016/j.jep.2024.118152 38614260

[B38] Dorn G. W. (2020). Mitofusins as mitochondrial anchors and tethers. J. Mol. Cell Cardiol. 142, 146–153. 10.1016/j.yjmcc.2020.04.016 32304672 PMC7275906

[B39] Evans C. S. Holzbaur E. L. F. (2020). Lysosomal degradation of depolarized mitochondria is rate-limiting in OPTN-dependent neuronal mitophagy. Autophagy 16 (5), 962–964. 10.1080/15548627.2020.1734330 32131674 PMC7144876

[B40] Feng J. Chen X. Shen J. (2017). Reactive nitrogen species as therapeutic targets for autophagy: implication for ischemic stroke. Expert Opin. Ther. Targets 21 (3), 305–317. 10.1080/14728222.2017.1281250 28081644

[B41] Frison M. Lockey B. S. Nie Y. Golder Z. Theiaspra E. Ryall C. D. (2025). Ubiquitin-mediated mitophagy regulates the inheritance of mitochondrial DNA mutations. Science 390 (6769), 156–163. 10.1126/science.adr5438 41066576

[B42] Gan Z. Y. Callegari S. Cobbold S. A. Cotton T. R. Mlodzianoski M. J. Schubert A. F. (2022). Activation mechanism of PINK1. Nature 602 (7896), 328–335. 10.1038/s41586-021-04340-2 34933320 PMC8828467

[B43] Ge Y. Shi X. Boopathy S. McDonald J. Smith A. W. Chao L. H. (2020). Two forms of Opa1 cooperate to complete fusion of the mitochondrial inner-membrane. Elife 9, e50973. 10.7554/eLife.50973 31922487 PMC7299343

[B44] Ge S. Wang L. Jin C. Xie H. Zheng G. Cui Z. (2024). Unveiling the neuroprotection effects of volvalerenic acid A: mitochondrial fusion induction via IDO1-mediated Stat3-Opa1 signaling pathway. Phytomedicine 129, 155555. 10.1016/j.phymed.2024.155555 38579641

[B45] Gong M. Guo Y. Dong H. Wu F. He Q. Gong J. (2024). Modified hu-lu-ba-wan protects diabetic glomerular podocytes via promoting PKM2-mediated mitochondrial dynamic homeostasis. Phytomedicine 123, 155247. 10.1016/j.phymed.2023.155247 38128393

[B46] Gray A. P. Chung E. Hsu R. L. Araki D. T. Hayoon A. G. Weaver N. D. (2025). Global, regional, and national sepsis incidence and mortality, 1990–2021: a systematic analysis. Lancet Glob. Health 13 (12), e2013–e2026. 10.1016/S1474-4422(24)00038-3 41135560

[B47] Griffin E. E. Detmer S. A. Chan D. C. (2006). Molecular mechanism of mitochondrial membrane fusion. Biochimica Biophysica Acta (BBA) - Mol. Cell Res. 1763 (5), 482–489. 10.1016/j.bbamcr.2006.02.003 16571363

[B48] Gugneja S. Virbasius C. M. Scarpulla R. C. (1996). Nuclear respiratory factors 1 and 2 utilize similar glutamine-containing clusters of hydrophobic residues to activate transcription. Mol. Cell Biol. 16 (10), 5708–5716. 10.1128/MCB.16.10.5708 8816484 PMC231571

[B49] Guo J. Miao Y. Nie F. Gao F. Li H. Wang Y. (2023). Zn-Shik-PEG nanoparticles alleviate inflammation and multi-organ damage in sepsis. J. Nanobiotechnol 21 (1), 448. 10.1186/s12951-023-02224-3 PMC10675904 38001490

[B50] Guo Q. Q. Wang S. S. Jiang X. Y. Xie X. C. Zou Y. Liu J. W. (2025). Mitochondrial ROS triggers mitophagy through activating the DNA damage response signaling pathway. Proc. Natl. Acad. Sci. 122 (40), e2502841122. 10.1073/pnas.2502841122 PMC12519191 41026812

[B51] Haileselassie B. Mukherjee R. Joshi A. U. Napier B. A. Massis L. M. Ostberg N. P. (2019). DRP1/Fis1 interaction mediates mitochondrial dysfunction in septic cardiomyopathy. J. Mol. Cell. Cardiol. 130, 160–169. 10.1016/j.yjmcc.2019.04.006 30981733 PMC6948926

[B52] Han X. Xu T. Fang Q. Zhang H. Yue L. Hu G. (2021). Quercetin hinders microglial activation to alleviate neurotoxicity via the interplay between NLRP3 inflammasome and mitophagy. Redox Biol. 44, 102010. 10.1016/j.redox.2021.102010 34082381 PMC8182123

[B53] Højlund K. Mogensen M. Sahlin K. Beck-Nielsen H. (2008). Mitochondrial dysfunction in type 2 diabetes and obesity. Endocrinol. Metab. Clin. North Am. 37 (3), 713–731. 10.1016/j.ecl.2008.06.006 18775360

[B54] Hou D. Liu R. Hao S. Dou Y. Chen G. Liu L. (2024a). Notoginsenoside R1 improves intestinal microvascular functioning in sepsis by targeting DRP1-mediated mitochondrial quality imbalance. Pharm. Biol. 62 (1), 250–260. 10.1080/13880209.2024.2318349 PMC10896147 38389274

[B55] Hou D. Liao H. Hao S. Liu R. Huang H. Duan C. (2024b). Curcumin simultaneously improves mitochondrial dynamics and myocardial cell bioenergy after sepsis via the SIRT1-DRP1/PGC-1α pathway. Heliyon 10 (7), e28501. 10.1016/j.heliyon.2024.e28501 PMC10998060 38586339

[B56] Hu D. Sheeja Prabhakaran H. Zhang Y. Y. Luo G. He W. Liou Y. C. (2024). Mitochondrial dysfunction in sepsis: mechanisms and therapeutic perspectives. Crit. Care 28, 292. 10.1186/s13054-024-05069-w 39227925 PMC11373266

[B57] Huang Q. Li J. Chen J. Zhang Z. Xu P. Qi H. (2023). Ginsenoside compound K protects against cerebral ischemia/reperfusion injury via Mul1/MFN2-mediated mitochondrial dynamics and bioenergy. J. Ginseng Res. 47 (3), 408–419. 10.1016/j.jgr.2022.10.004 37252276 PMC10214289

[B58] Huang S. J. Ma D. F. Yu C. Li J. Tu X. Huang Z. (2025). A special latch in yeast mitofusin guarantees mitochondrial fusion by stabilizing self-assembly. Nat. Commun. 16 (1), 9644. 10.1038/s41467-025-64646-x 41173874 PMC12578975

[B59] Huang R. Zhang C. Tang Y. Li X. (2025). Baoshen rehabilitation granules ameliorate sepsis-induced acute kidney injury by modulating oxidative stress and mitochondrial damage. Toxicol. Res. 14 (4), tfaf087. 10.1093/toxres/tfaf087 PMC12257101 40666292

[B60] Huang S. Zeng Z. Chu Y. Zhang S. Zhou J. Hu Z. (2025). Mitigation of lipopolysaccharide-induced intestinal injury in rats by *Chimonanthus nitens* oliv. essential oil via suppression of mitochondrial fusion protein mitofusin 2 (MFN2)-mediated mitochondrial-associated endoplasmic reticulum membranes (MAMs) formation. J. Ethnopharmacol. 337 (Pt 2), 118856. 10.1016/j.jep.2024.118856 39332614

[B61] Huang P. Wang Q. Zhu Y. Hu X. Fang X. Deng W. (2026). Qiang-Xin 1 formula improves cardiac function and alleviates myocardial injury in lipopolysaccharide-induced septic mice by activating calcium/calmodulin-dependent protein kinase I-mediated mitophagy. J. Ethnopharmacol. 358, 121033. 10.1016/j.jep.2025.121033 41397542

[B62] Iba T. Helms J. Maier C. L. Ferrer R. Levy J. H. (2024). Autophagy and autophagic cell death in sepsis: friend or foe? J. Intensive Care 12, 41. 10.1186/s40560-024-00754-y 39449054 PMC11520123

[B64] Jin M. Lazarou M. Wang C. Kane L. A. Narendra D. P. Youle R. J. (2010). Mitochondrial membrane potential regulates PINK1 import and proteolytic destabilization by PARL. J. Cell Biol. 191 (5), 933–942. 10.1083/jcb.201008084 21115803 PMC2995166

[B65] Jin X. Wang J. Gao K. Zhang P. Yao L. Tang Y. (2017). Dysregulation of INF2-mediated mitochondrial fission in SPOP-mutated prostate cancer. PLOS Genet. 13 (4), e1006748. 10.1371/journal.pgen.1006748 28448495 PMC5426793

[B66] Jin K. Ma Y. Manrique-Caballero C. L. Li H. Emlet D. R. Li S. (2020). Activation of AMP-activated protein kinase during sepsis/inflammation improves survival by preserving cellular metabolic fitness. FASEB J. 34 (5), 7036–7057. 10.1096/fj.201901900R 32246808 PMC11956121

[B67] Jun M. S. Kim H. S. Kim Y. M. Kim H. J. Park E. J. Lee J. H. (2012). Ethanol extract of Prunella vulgaris var. lilacina inhibits HMGB1 release by induction of heme oxygenase-1 in LPS-activated RAW 264.7 cells and CLP-induced septic mice. Phytother. Res. 26 (4), 605–612. 10.1002/ptr.3613 21971692

[B68] Kage F. Vicente-Manzanares M. McEwan B. C. Kettenbach A. N. Higgs H. N. (2022). Myosin II proteins are required for organization of calcium-induced actin networks upstream of mitochondrial division. MBoC 33 (7), ar63. 10.1091/mbc.E22-01-0005 35427150 PMC9561854

[B69] Kameoka S. Adachi Y. Okamoto K. Iijima M. Sesaki H. (2018). Phosphatidic acid and cardiolipin coordinate mitochondrial dynamics. Trends Cell Biol. 28 (1), 67–76. 10.1016/j.tcb.2017.08.011 28911913 PMC5742555

[B70] Kazi N. H. Klink N. Gallant K. Kipka G. M. Gersch M. (2025). Chimeric deubiquitinase engineering reveals structural basis for specific inhibition of the mitophagy regulator USP30. Nat. Struct. Mol. Biol. 32 (9), 1776–1786. 10.1038/s41594-025-01534-4 40325251 PMC12440824

[B71] Kazlauskaite A. Kondapalli C. Gourlay R. Campbell D. G. Ritorto M. S. Hofmann K. (2014). Parkin is activated by PINK1-dependent phosphorylation of ubiquitin at Ser65. Biochem. J. 460 (Pt 1), 127–139. 10.1042/BJ20140334 24660806 PMC4000136

[B72] Kim J. Kundu M. Viollet B. Guan K. L. (2011). AMPK and mTOR regulate autophagy through direct phosphorylation of Ulk1. Nat. Cell Biol. 13 (2), 132–141. 10.1038/ncb2152 21258367 PMC3987946

[B73] Kim S. K. Joe Y. Zheng M. Kim H. J. Yu J. K. Cho G. J. (2014). Resveratrol induces hepatic mitochondrial biogenesis through the sequential activation of nitric oxide and carbon monoxide production. Antioxid. Redox Signal 20 (16), 2589–2605. 10.1089/ars.2012.5138 24041027 PMC4024846

[B74] Korobova F. Ramabhadran V. Higgs H. N. (2013). An actin-dependent step in mitochondrial fission mediated by the ER-associated formin INF2. Science 339 (6118), 464–467. 10.1126/science.1228360 23349293 PMC3843506

[B75] Koyano F. Okatsu K. Kosako H. Tamura Y. Go E. Kimura M. (2014). Ubiquitin is phosphorylated by PINK1 to activate parkin. Nature 510 (7503), 162–166. 10.1038/nature13392 24784582

[B76] Kuang Y. Ma K. Zhou C. Ding P. Zhu Y. Chen Q. (2016). Structural basis for the phosphorylation of FUNDC1 LIR as a molecular switch of mitophagy. Autophagy 12 (12), 2363–2373. 10.1080/15548627.2016.1238552 27653272 PMC5173264

[B77] Kuang J. Fang J. Hu S. Yang X. Fan X. (2024). Mechanism of microrna-218-5P in mitochondrial biogenesis of sepsis-induced acute kidney injury by the regulation of PGC-1Α. Shock 62 (3), 426–436. 10.1097/SHK.0000000000002410 38888503

[B78] Kumar A. Aguirre J. D. Condos T. E. Martinez-Torres R. J. Chaugule V. K. Toth R. (2015). Disruption of the autoinhibited state primes the E3 ligase parkin for activation and catalysis. EMBO J. 34 (20), 2506–2521. 10.15252/embj.201592337 26254304 PMC4609183

[B79] Lagouge M. Argmann C. Gerhart-Hines Z. Meziane H. Lerin C. Daussin F. (2006). Resveratrol improves mitochondrial function and protects against metabolic disease by activating SIRT1 and PGC-1alpha. Cell 127 (6), 1109–1122. 10.1016/j.cell.2006.11.013 17112576

[B80] Lai B. Gu M. Sun J. Zhang J. (2025). Targeting mitochondria: unveiling novel therapeutic frontiers in sepsis. Int. Immunopharmacol. 161, 115078. 10.1016/j.intimp.2025.115078 40505227

[B81] Larson C. Opichka M. McGlynn M. L. Collins C. W. Slivka D. (2020). Exercise- and cold-induced Human PGC-1α mRNA isoform specific responses. Int. J. Environ. Res. Public Health 17 (16), 5740. 10.3390/ijerph17165740 32784428 PMC7460212

[B82] Lazarou M. Sliter D. A. Kane L. A. Sarraf S. A. Wang C. Burman J. L. (2015). The ubiquitin kinase PINK1 recruits autophagy receptors to induce mitophagy. Nature 524 (7565), 309–314. 10.1038/nature14893 26266977 PMC5018156

[B83] Lee H. C. Wei Y. H. (2005). Mitochondrial biogenesis and mitochondrial DNA maintenance of mammalian cells under oxidative stress. Int. J. Biochem. & Cell Biol. 37 (4), 822–834. 10.1016/j.biocel.2004.09.010 15694841

[B84] Lee H. Yoon Y. (2018). Mitochondrial membrane dynamics—functional positioning of OPA1. Antioxidants (Basel) 7 (12), 186. 10.3390/antiox7120186 30544804 PMC6316456

[B85] Lelubre C. Vincent J. L. (2018). Mechanisms and treatment of organ failure in sepsis. Nat. Rev. Nephrol. 14 (7), 417–427. 10.1038/s41581-018-0005-7 29691495

[B86] Lemasters J. J. Mary A. L. Larchmont M. A. (2005). Selective mitochondrial autophagy, or mitophagy, as a targeted defense against oxidative stress, mitochondrial dysfunction, and aging. Rejuvenation Res. 8 (1), 3–5. 10.1089/rej.2005.8.3 15798367

[B87] Li C. Ding X. Y. Xiang D. M. Xu J. Huang X. L. Hou F. F. (2015). Enhanced M1 and impaired M2 macrophage polarization and reduced mitochondrial biogenesis *via* inhibition of AMP kinase in chronic kidney disease. Cell Physiol. Biochem. 36 (1), 358–372. 10.1159/000430106 25967974

[B88] Li X. Zhang Y. Yu J. Mu R. Wu L. Shi J. (2018). Activation of protein kinase C-α/heme oxygenase-1 signaling pathway improves mitochondrial dynamics in lipopolysaccharide-activated NR8383 cells. Exp. Ther. Med. 16 (2), 1529–1537. 10.3892/etm.2018.6290 30112072 PMC6090414

[B89] Li J. Lin Q. Shao X. Li S. Zhu X. Wu J. (2023). HIF1α-BNIP3-mediated mitophagy protects against renal fibrosis by decreasing ROS and inhibiting activation of the NLRP3 inflammasome. Cell Death Dis. 14 (3), 200. 10.1038/s41419-023-05587-5 36928344 PMC10020151

[B90] Li H. Liu Q. Zhu C. Sun X. Sun C. Yu C. (2023). β-Nicotinamide mononucleotide activates NAD+/SIRT1 pathway and attenuates inflammatory and oxidative responses in the hippocampus regions of septic mice. Redox Biol. 63, 102745. 10.1016/j.redox.2023.102745 37201414 PMC10206198

[B91] Li Z. Pan H. Yang J. Chen D. Wang Y. Zhang H. (2023). Xuanfei Baidu formula alleviates impaired mitochondrial dynamics and activated NLRP3 inflammasome by repressing NF-κB and MAPK pathways in LPS-induced ALI and inflammation models. Phytomedicine 108, 154545. 10.1016/j.phymed.2022.154545 36423572 PMC9643338

[B92] Li X. Wang S. Luo M. Wang M. Wu S. Liu C. (2024). Carnosol alleviates sepsis-induced pulmonary endothelial barrier dysfunction by targeting nuclear factor erythroid2-related factor 2/sirtuin-3 signaling pathway to attenuate oxidative damage. Phytother. Res. 38 (5), 2182–2197. 10.1002/ptr.8138 38414287

[B93] Liao X. Wang Y. Zhang Z. Qu X. Zhou G. (2026). Bidirectional regulation of NLRP3 inflammasome and mitochondrial quality control in sepsis: mechanisms and therapeutic implications. Mediat. Inflamm. 2026, 3168669. 10.1155/mi/3168669 PMC12848910 41613015

[B94] Lira Chavez F. M. Gartzke L. P. van Beuningen F. E. Wink S. E. Henning R. H. Krenning G. (2023). Restoring the infected powerhouse: mitochondrial quality control in sepsis. Redox Biol. 68, 102968. 10.1016/j.redox.2023.102968 38039825 PMC10711241

[B95] Liu R. Chan D. C. (2017). OPA1 and cardiolipin team up for mitochondrial fusion. Nat. Cell Biol. 19 (7), 760–762. 10.1038/ncb3565 28628085 PMC5757513

[B96] Liu L. Feng D. Chen G. Chen M. Zheng Q. Song P. (2012). Mitochondrial outer-membrane protein FUNDC1 mediates hypoxia-induced mitophagy in mammalian cells. Nat. Cell Biol. 14 (2), 177–185. 10.1038/ncb2422 22267086

[B97] Liu J. Yang C. Zhang W. Su H. Liu Z. Pan Q. (2019). Disturbance of mitochondrial dynamics and mitophagy in sepsis-induced acute kidney injury. Life Sci. 235, 116828. 10.1016/j.lfs.2019.116828 31479679

[B98] Liu A. Kage F. Abdulkareem A. F. Aguirre-Huamani M. P. Sapp G. Aydin H. (2024). Fatty acyl-coenzyme A activates mitochondrial division through oligomerization of MiD49 and MiD51. Nat. Cell Biol. 26 (5), 731–744. 10.1038/s41556-024-01400-3 PMC11404400 38594588

[B99] Liu Y. Zhang Y. You G. Zheng D. He Z. Guo W. (2024). Tangeretin attenuates acute lung injury in septic mice by inhibiting ROS-mediated NLRP3 inflammasome activation via regulating PLK1/AMPK/DRP1 signaling axis. Inflamm. Res. 73 (1), 47–63. 10.1007/s00011-023-01819-8 38147126

[B100] Liu J. Le Y. Wang J. Zheng J. Yuan A. Guo J. (2025). Fruit of *Physalis angulata* L. and anti-inflammatory potential: an *in silico,*, *in vitro,*, and *in vivo* study focusing on PFKFB3. Phytomedicine 143, 156813. 10.1016/j.phymed.2025.156813 40382942

[B101] Ljubicic V. Hood D. A. (2009). Diminished contraction-induced intracellular signaling towards mitochondrial biogenesis in aged skeletal muscle. Aging Cell 8 (4), 394–404. 10.1111/j.1474-9726.2009.00483.x 19416128

[B102] Long Y. Li Y. Ma Z. Xie Y. Zhao H. Zhang M. (2025). Epimedii Folium and Ligustri Lucidi Fructus synergistically delay renal aging through AMPK/ULK1/Bcl2L13-mediated mitophagy. J. Ethnopharmacol. 346, 119668. 10.1016/j.jep.2025.119668 40122318

[B103] Losón O. C. Song Z. Chen H. Chan D. C. (2013). Fis1, Mff, MiD49, and MiD51 mediate DRP1 recruitment in mitochondrial fission. Mol. Biol. Cell 24 (5), 659–667. 10.1091/mbc.E12-10-0721 23283981 PMC3583668

[B104] Luo P. An Y. He J. Xing X. Zhang Q. Liu X. (2024). Icaritin with autophagy/mitophagy inhibitors synergistically enhances anticancer efficacy and apoptotic effects through PINK1/Parkin-mediated mitophagy in hepatocellular carcinoma. Cancer Lett. 587, 216621. 10.1016/j.canlet.2024.216621 38242198

[B105] Lv M. Wang C. Li F. Peng J. Wen B. Gong Q. (2017). Structural insights into the recognition of phosphorylated FUNDC1 by LC3B in mitophagy. Protein Cell 8 (1), 25–38. 10.1007/s13238-016-0328-8 27757847 PMC5233613

[B106] Mariotto S. Esposito E. Di Paola R. Ciampa A. Mazzon E. de Prati A. C. (2008). Protective effect of Arbutus unedo aqueous extract in carrageenan-induced lung inflammation in mice. Pharmacol. Res. 57 (2), 110–124. 10.1016/j.phrs.2007.12.005 18249557

[B107] Ma K. Zhang Z. Chang R. Cheng H. Mu C. Zhao T. (2020). Dynamic PGAM5 multimers dephosphorylate BCL-xL or FUNDC1 to regulate mitochondrial and cellular fate. Cell Death Differ. 27 (3), 1036–1051. 10.1038/s41418-019-0396-4 31367011 PMC7206082

[B108] Marchesan E. Nardin A. Mauri S. Bernardo G. Chander V. Di Paola S. (2024). Activation of Ca2+ phosphatase calcineurin regulates Parkin translocation to mitochondria and mitophagy in flies. Cell Death Differ. 31 (2), 217–238. 10.1038/s41418-023-01251-9 38238520 PMC10850161

[B109] McLaughlin K. L. McClung J. M. Fisher-Wellman K. H. (2018). Bioenergetic consequences of compromised mitochondrial DNA repair in the mouse heart. Biochem. Biophys. Res. Commun. 504, 742–748. 10.1016/j.bbrc.2018.09.022 30217445 PMC6173612

[B110] Mesmin B. (2016). Mitochondrial lipid transport and biosynthesis: a complex balance. J. Cell Biol. 214 (1), 9–11. 10.1083/jcb.201606069 27354376 PMC4932376

[B111] Mokhtari B. Yavari R. Badalzadeh R. Mahmoodpoor A. (2022). An overview on mitochondrial-based therapies in sepsis-related myocardial dysfunction: mitochondrial transplantation as a promising approach. Can. J. Infect. Dis. Med. Microbiol. 2022, 3277274. 10.1155/2022/3277274 35706715 PMC9192296

[B112] Moore A. S. Holzbaur E. L. (2016). Dynamic recruitment and activation of ALS-associated TBK1 with its target optineurin are required for efficient mitophagy. Proc. Natl. Acad. Sci. U. S. A. 113 (24), E3349–E3358. 10.1073/pnas.1523810113 27247382 PMC4914160

[B113] Murakawa T. Yamaguchi O. Hashimoto A. Hikoso S. Takeda T. Oka T. (2015). Bcl-2-like protein 13 is a mammalian Atg32 homologue that mediates mitophagy and mitochondrial fragmentation. Nat. Commun. 6, 7527. 10.1038/ncomms8527 26146385 PMC4501433

[B114] Naón D. Hernández-Alvarez M. I. Shinjo S. Wieczor M. Ivanova S. Martins de Brito O. (2023). Splice variants of mitofusin 2 shape the endoplasmic reticulum and tether it to mitochondria. Science 380 (6651), eadh9351. 10.1126/science.adh9351 37347868

[B115] Nemet M. Karakus I. S. Gmehlin G. C. Alves Halpern G. Ghafouri K. Abramovich S. (2025). Mortality in the Sepsis-3 era: a systematic review of multicenter randomized controlled trials. Shock. 10.1097/SHK.0000000000002687 40997273

[B116] Neupert W. Herrmann J. M. (2007). Translocation of proteins into mitochondria. Annu. Rev. Biochem. 76, 723–749. 10.1146/annurev.biochem.76.052705.163409 17263664

[B117] Ni H. Y. Yu L. Zhao X. L. Wang L. Zhao C. Huang H. (2022). Seed oil of Rosa roxburghii tratt against non-alcoholic fatty liver disease *in vivo* and *in vitro* through PPARα/PGC-1α-mediated mitochondrial oxidative metabolism. Phytomedicine 98, 153919. 10.1016/j.phymed.2021.153919 35104757

[B118] Noh S. Phorl S. Naskar R. Oeum K. Seo Y. Kim E. (2020). p32/C1QBP regulates OMA1-dependent proteolytic processing of OPA1 to maintain mitochondrial connectivity related to mitochondrial dysfunction and apoptosis. Sci. Rep. 10 (1), 10618. 10.1038/s41598-020-67457-w 32606429 PMC7327069

[B119] Novak I. Kirkin V. McEwan D. G. Zhang J. Wild P. Rozenknop A. (2010). Nix is a selective autophagy receptor for mitochondrial clearance. EMBO Rep. 11 (1), 45–51. 10.1038/embor.2009.256 20010802 PMC2816619

[B120] Okatsu K. Oka T. Iguchi M. Imamura K. Kosako H. Tani N. (2012). PINK1 autophosphorylation upon membrane potential dissipation is essential for Parkin recruitment to damaged mitochondria. Nat. Commun. 3 (1), 1016. 10.1038/ncomms2016 22910362 PMC3432468

[B121] Ong S. B. Subrayan S. Lim S. Y. Yellon D. M. Davidson S. M. Hausenloy D. J. (2010). Inhibiting mitochondrial fission protects the heart against ischemia/reperfusion injury. Circulation 121 (18), 2012–2022. 10.1161/CIRCULATIONAHA.109.906610 20421521

[B122] Onishi M. Yamano K. Sato M. Matsuda N. Okamoto K. (2021). Molecular mechanisms and physiological functions of mitophagy. EMBO J. 40 (3), e104705. 10.15252/embj.2020104705 33438778 PMC7849173

[B123] Otera H. Wang C. Cleland M. M. Setoguchi K. Yokota S. Youle R. J. (2010). Mff is an essential factor for mitochondrial recruitment of DRP1 during mitochondrial fission in mammalian cells. J. Cell Biol. 191 (6), 1141–1158. 10.1083/jcb.201007152 21149567 PMC3002033

[B124] Otsu K. Murakawa T. Yamaguchi O. (2015). BCL2L13 is a mammalian homolog of the yeast mitophagy receptor Atg32. Autophagy 11 (10), 1932–1933. 10.1080/15548627.2015.1084459 26506896 PMC4824574

[B125] Padman B. S. Nguyen T. N. Uoselis L. Skulsuppaisarn M. Nguyen L. K. Lazarou M. (2019). LC3/GABARAPs drive ubiquitin-independent recruitment of optineurin and NDP52 to amplify mitophagy. Nat. Commun. 10 (1), 408. 10.1038/s41467-019-08335-6 30679426 PMC6345886

[B126] Paik S. Kim J. K. Shin H. J. Park E. J. Kim I. S. Jo E. K. (2025). Updated insights into the molecular networks for NLRP3 inflammasome activation. Cell Mol. Immunol. 22 (6), 563–596. 10.1038/s41423-025-01284-9 40307577 PMC12125403

[B127] Pan D. Chen P. Zhang H. Zhao Q. Fang W. Ji S. (2025). Mitochondrial quality control: a promising target of traditional Chinese medicine in the treatment of cardiovascular disease. Pharmacol. Res. 215, 107712. 10.1016/j.phrs.2025.107712 40154932

[B128] Panicker N. Dawson V. L. Dawson T. M. (2017). Activation mechanisms of the E3 ubiquitin ligase parkin. Biochem. J. 474 (18), 3075–3086. 10.1042/BCJ20170476 28860335

[B129] Patoli D. Mignotte F. Deckert V. Dusuel A. Dumont A. Rieu A. (2020). Inhibition of mitophagy drives macrophage activation and antibacterial defense during sepsis. J. Clin. Invest 130 (11), 5858–5874. 10.1172/JCI130996 32759503 PMC7598049

[B130] Patti M. E. Butte A. J. Crunkhorn S. Cusi K. Berria R. Kashyap S. (2003). Coordinated reduction of genes of oxidative metabolism in humans with insulin resistance and diabetes: potential role of PGC1 and NRF1. Proc. Natl. Acad. Sci. 100 (14), 8466–8471. 10.1073/pnas.1032913100 12832613 PMC166252

[B131] Paunikar S. Chakole V. (2024). Hyperoxia in sepsis and septic shock: a comprehensive review of clinical evidence and therapeutic implications. Cureus 16 (9), e68597. 10.7759/cureus.68597 39371803 PMC11452320

[B132] Perry H. M. Huang L. Wilson R. J. Bajwa A. Sesaki H. Yan Z. (2018). Dynamin-related protein 1 deficiency promotes recovery from AKI. J. Am. Soc. Nephrol. 29 (1), 194–206. 10.1681/ASN.2017060659 29084809 PMC5748924

[B133] Pu K. Yang S. Sheng R. Chen J. Dai Y. Wood I. C. (2025). Chuanxiong-Danggui herb pair alleviated cognitive deficits of APP/PS1 mice by promoting mitophagy. J. Ethnopharmacol. 350, 119988. 10.1016/j.jep.2025.119988 40389086

[B134] Qi Y. Rajbanshi B. Hao R. Dang Y. Xu C. Lu W. (2025). The dual role of PGAM5 in inflammation. Exp. Mol. Med. 57 (2), 298–311. 10.1038/s12276-025-01391-7 PMC11873181 39930129

[B135] Qi W. Yang L. Pan Y. Lv K. Zhao L. Zhang Y. (2025). Chinese leek-derived extracellular vesicles ameliorate sarcopenia by regulating mitochondrial biogenesis and autophagy via AMPK and maintaining myosin homeostasis. J. Nanobiotechnol 23 (1), 721. 10.1186/s12951-025-03764-6 PMC12632053 41261435

[B136] Qian L. Zhu Y. Deng C. Liang Z. Chen J. Chen Y. (2024). Peroxisome proliferator-activated receptor gamma coactivator-1 (PGC-1) family in physiological and pathophysiological process and diseases. Sig Transduct. Target Ther. 9 (1), 50. 10.1038/s41392-024-01756-w PMC10904817 38424050

[B137] Cheng Q. Wan Y. Yang W. Tian M. Wang Y. He H. (2020). Gastrodin protects H9c2 cardiomyocytes against oxidative injury by ameliorating imbalanced mitochondrial dynamics and mitochondrial dysfunction. Acta Pharmacol. Sin. 41 (10), 1314–1327. 10.1038/s41401-020-0382-x 32203078 PMC7608121

[B138] Qin X. Zhao Y. Gong J. Huang W. Su H. Yuan F. (2019). Berberine protects glomerular podocytes via inhibiting DRP1-Mediated mitochondrial fission and dysfunction. Theranostics 9 (6), 1698–1713. 10.7150/thno.30640 31037132 PMC6485199

[B139] Rodgers J. T. Lerin C. Haas W. Gygi S. P. Spiegelman B. M. Puigserver P. (2005). Nutrient control of glucose homeostasis through a complex of PGC-1α and SIRT1. Nature 434 (7029), 113–118. 10.1038/nature03354 15744310

[B140] Roperto S. De Falco F. Perillo A. Catoi C. Roperto F. (2019). Mitophagy mediated by BNIP3 and BNIP3L/NIX in urothelial cells of the urinary bladder of cattle harbouring bovine papillomavirus infection. Veterinary Microbiol. 236, 108396. 10.1016/j.vetmic.2019.108396 31500722

[B141] Rosa F. L. L. de Souza I. I. A. Monnerat G. Campos de Carvalho A. C. Maciel L. (2023). Aging triggers mitochondrial dysfunction in mice. Int. J. Mol. Sci. 24 (13), 10591. 10.3390/ijms241310591 37445770 PMC10342046

[B142] Samant S. A. Zhang H. J. Hong Z. Pillai V. B. Sundaresan N. R. Wolfgeher D. (2014). SIRT3 deacetylates and activates OPA1 to regulate mitochondrial dynamics during stress. Mol. Cell Biol. 34 (5), 807–819. 10.1128/MCB.01483-13 24344202 PMC4023816

[B143] Sauvé V. Sung G. Soya N. Kozlov G. Blaimschein N. Miotto L. S. (2018). Mechanism of parkin activation by phosphorylation. Nat. Struct. Mol. Biol. 25 (7), 623–630. 10.1038/s41594-018-0088-7 29967542

[B144] Scarpulla R. C. Vega R. B. Kelly D. P. (2012). Transcriptional integration of mitochondrial biogenesis. Trends Endocrinol. & Metabolism 23 (9), 459–466. 10.1016/j.tem.2012.06.006 22817841 PMC3580164

[B145] Schweers R. L. Zhang J. Randall M. S. Loyd M. R. Li W. Dorsey F. C. (2007). NIX is required for programmed mitochondrial clearance during reticulocyte maturation. Proc. Natl. Acad. Sci. 104 (49), 19500–19505. 10.1073/pnas.0708818104 18048346 PMC2148318

[B146] Shahannaz D. C. Sugiura T. Ferrell B. E. Yoshida T. (2026). Engineering mitochondrial biogenesis in iPSC-CMs: CRISPR-guided approaches for advanced cardiomyocyte development. J. Cardiovasc Dev. Dis. 13 (2), 77. 10.3390/jcdd13020077 41745325 PMC12942445

[B147] Shpilka T. Du Y. Yang Q. Melber A. Uma Naresh N. Lavelle J. (2021). UPRmt scales mitochondrial network expansion with protein synthesis *via* mitochondrial import in *Caenorhabditis elegans* . Nat. Commun. 12 (1), 479. 10.1038/s41467-020-20784-y 33473112 PMC7817664

[B148] Singer M. Deutschman C. S. Seymour C. W. Shankar-Hari M. Annane D. Bauer M. (2016). The third international consensus definitions for sepsis and septic shock (Sepsis-3). JAMA 315 (8), 801–810. 10.1001/jama.2016.0287 26903338 PMC4968574

[B149] Sloat S. R. Whitley B. N. Engelhart E. A. Hoppins S. (2019). Identification of a mitofusin specificity region that confers unique activities to Mfn1 and MFN2. Mol. Biol. Cell 30 (17), 2309–2319. 10.1091/mbc.E19-05-0291 31188717 PMC6743458

[B150] Song Z. Chen H. Fiket M. Alexander C. Chan D. C. (2007). OPA1 processing controls mitochondrial fusion and is regulated by mRNA splicing, membrane potential, and Yme1L. J. Cell Biol. 178 (5), 749–755. 10.1083/jcb.200704110 17709429 PMC2064540

[B151] Song Y. Lin W. Zhu W. (2023). Traditional Chinese medicine for treatment of sepsis and related multi-organ injury. Front. Pharmacol. 14, 1003658. 10.3389/fphar.2023.1003658 36744251 PMC9892725

[B152] Song Z. Li J. Gong X. (2025). Dahuang chuanxiong decoction against contrast-induced nephropathy: multi-Omics, crosstalk between BNIP3-mediated mitophagy and IL-17 pathway. Phytomedicine 138, 156416. 10.1016/j.phymed.2025.156416 39889489

[B153] Taguchi N. Ishihara N. Jofuku A. Oka T. Mihara K. (2007). Mitotic phosphorylation of dynamin-related GTPase DRP1 participates in mitochondrial fission. J. Biol. Chem. 282 (15), 11521–11529. 10.1074/jbc.M607279200 17301055

[B154] Tábara L. C. Burr S. P. Frison M. Chowdhury S. R. Paupe V. Nie Y. (2024). MTFP1 controls mitochondrial fusion to regulate inner membrane quality control and maintain mtDNA levels. Cell 187 (14), 3619–3637.e27. 10.1016/j.cell.2024.05.017 38851188

[B155] Tang M. Rong D. Gao X. Lu G. Tang H. Wang P. (2025). A positive feedback loop between SMAD3 and PINK1 in regulation of mitophagy. Cell Discov. 11 (1), 22. 10.1038/s41421-025-00774-4 40064862 PMC11894195

[B156] Tang R. Qin S. Xu Q. Lin W. Zhang S. Peng Y. (2025). Lipopolysaccharide-induced histone lactylation mediates m6A RNA modification causing mitochondrial dysfunction and pulmonary fibroblasts activation to exacerbate sepsis-associated pulmonary fibrosis. Respir. Res. 26 (1), 347. 10.1186/s12931-025-03422-3 PMC12709686 41402767

[B157] Thatavarthy S. Abriata L. A. Meireles F. T. P. Zuccaro K. E. Gargey Iragavarapu A. Sullivan G. M. (2025). Cardiolipin dynamics promote membrane remodeling by mitochondrial OPA1. Nat. Commun. 16 (1), 8685. 10.1038/s41467-025-63813-4 41027961 PMC12484737

[B158] Tilokani L. Nagashima S. Paupe V. Prudent J. (2018). Mitochondrial dynamics: overview of molecular mechanisms. Essays Biochem. 62 (3), 341–360. 10.1042/EBC20170104 30030364 PMC6056715

[B159] Tran M. Tam D. Bardia A. Bhasin M. Rowe G. C. Kher A. (2011). PGC-1α promotes recovery after acute kidney injury during systemic inflammation in mice. J. Clin. Invest 121 (10), 4003–4014. 10.1172/JCI58662 21881206 PMC3195479

[B160] Tyrrell D. J. Goldstein D. R. (2021). Ageing and atherosclerosis: vascular intrinsic and extrinsic factors and potential role of IL-6. Nat. Rev. Cardiol. 18 (1), 58–68. 10.1038/s41569-020-0431-7 32918047 PMC7484613

[B161] Ventura-Clapier R. Garnier A. Veksler V. (2008). Transcriptional control of mitochondrial biogenesis: the central role of PGC-1α. Cardiovasc Res. 79 (2), 208–217. 10.1093/cvr/cvn098 18430751

[B162] Wang L. Cho Y. L. Tang Y. Wang J. Park J. E. Wu Y. (2018). PTEN-L is a novel protein phosphatase for ubiquitin dephosphorylation to inhibit PINK1–Parkin-mediated mitophagy. Cell Res. 28 (8), 787–802. 10.1038/s41422-018-0056-0 29934616 PMC6082900

[B163] Wang X. Shen K. Wang J. Liu K. Wu G. Li Y. (2020). Hypoxic preconditioning combined with curcumin promotes cell survival and mitochondrial quality of bone marrow mesenchymal stem cells, and accelerates cutaneous wound healing via PGC-1α/SIRT3/HIF-1α signaling. Free Radic. Biol. Med. 159, 164–176. 10.1016/j.freeradbiomed.2020.07.023 32745765

[B164] Wang R. Mishra P. Garbis S. D. Moradian A. Sweredoski M. J. Chan D. C. (2021). Identification of new OPA1 cleavage site reveals that short isoforms regulate mitochondrial fusion. Mol. Biol. Cell 32 (2), 157–168. 10.1091/mbc.E20-09-0605 33237841 PMC8120690

[B165] Wang X. Jiang Y. Zhang Y. Sun Q. Ling G. Jiang J. (2022). The roles of the mitophagy inducer Danqi pill in heart failure: a new therapeutic target to preserve energy metabolism. Phytomedicine 99, 154009. 10.1016/j.phymed.2022.154009 35217438

[B166] Wang M. Wang L. Zhou Y. Feng X. Ye C. Wang C. (2022). Shen Shuai II recipe attenuates renal fibrosis in chronic kidney disease by improving hypoxia-induced the imbalance of mitochondrial dynamics via PGC-1α activation. Phytomedicine 98, 153947. 10.1016/j.phymed.2022.153947 35104767

[B167] Wang S. Long H. Hou L. Feng B. Ma Z. Wu Y. (2023). The mitophagy pathway and its implications in human diseases. Signal Transduct. Target Ther. 8, 304. 10.1038/s41392-023-01503-7 37582956 PMC10427715

[B168] Wang J. Pu X. Zhuang H. Guo Z. Wang M. Yang H. (2025). Astragaloside IV alleviates septic myocardial injury through DUSP1-Prohibitin 2 mediated mitochondrial quality control and ER-autophagy. J. Adv. Res. 75, 561–580. 10.1016/j.jare.2024.10.030 39550027 PMC12789773

[B169] Wang Z. Wang Y. Dong C. Miao K. Jiang B. Zhou D. (2025). Po-Ge-Jiu-Xin decoction alleviate sepsis-induced cardiomyopathy via regulating phosphatase and tensin homolog-induced putative kinase 1/parkin-mediated mitophagy. J. Ethnopharmacol. 337, 118952. 10.1016/j.jep.2024.118952 39426573

[B170] Wang N. Wang X. Lan B. Gao Y. Cai Y. (2025). DRP1, fission and apoptosis. Cell Death Discov. 11 (1), 150. 10.1038/s41420-025-02458-0 40195359 PMC11977278

[B171] Wang Y. Wang W. Sun J. Zhou S. Ni L. Dong M. (2026). Exogenous pyruvate restores mitochondrial bioenergetics by synergizing with the AMPK-mTOR-SIRT3 pathway to alleviate sepsis-associated acute kidney injury. Chem. Biol. Interact. 434, 112089. 10.1016/j.cbi.2026.112089 42031090

[B172] Wauer T. Simicek M. Schubert A. Komander D. (2015a). Mechanism of phospho-ubiquitin induced Parkin activation. Nature 524 (7565), 370–374. 10.1038/nature14879 26161729 PMC4984986

[B173] Wauer T. Swatek K. N. Wagstaff J. L. Gladkova C. Pruneda J. N. Michel M. A. (2015b). Ubiquitin Ser65 phosphorylation affects ubiquitin structure, chain assembly and hydrolysis. EMBO J. 34 (3), 307–325. 10.15252/embj.201489847 25527291 PMC4339119

[B174] Wei X. Wang Y. Lao Y. Weng J. Deng R. Li S. (2023). Effects of honokiol protects against chronic kidney disease via BNIP3/NIX and FUNDC1-mediated mitophagy and AMPK pathways. Mol. Biol. Rep. 50 (8), 6557–6568. 10.1007/s11033-023-08592-1 37338733

[B175] Wen E. Cao Y. He S. Zhang Y. You L. Wang T. (2024). The mitochondria-targeted Kaempferol nanoparticle ameliorates severe acute pancreatitis. J. Nanobiotechnol. 22 (1), 148. 10.1186/s12951-024-02439-y PMC10993609 38570776

[B176] Wendrich K. Gallant K. Recknagel S. Petroulia S. Kazi N. H. Hane J. A. (2025). Discovery and mechanism of K63-linkage-directed deubiquitinase activity in USP53. Nat. Chem. Biol. 21 (5), 746–757. 10.1038/s41589-024-01777-0 PMC12037411 39587316

[B177] Wu Y. Yao Y. M. Ke H. L. Ying L. Wu Y. Zhao G. J. (2019). Mdivi-1 protects CD4+ T cells against apoptosis via balancing mitochondrial fusion-fission and preventing the induction of endoplasmic reticulum stress in sepsis. Mediat. Inflamm. 2019, 7329131. 10.1155/2019/7329131 31263382 PMC6541989

[B178] Xiao Z. Long J. Zhang J. Qiu Z. Zhang C. Liu H. (2023). Administration of protopine prevents mitophagy and acute lung injury in sepsis. Front. Pharmacol. 14, 1104185. 10.3389/fphar.2023.1104185 37361224 PMC10285494

[B179] Xie J. Wang H. Kang Y. Zhou L. Liu Z. Qin B. (2020). The epidemiology of sepsis in Chinese ICUs: a national cross-sectional survey. Crit. Care Med. 48 (3), e209–e218. 10.1097/CCM.0000000000004155 31804299

[B180] Xie L. Zhou C. Wu Y. Fu X. Zhang G. Han X. (2023). Wenqingyin suppresses ferroptosis in the pathogenesis of sepsis-induced liver injury by activating the Nrf2-mediated signaling pathway. Phytomedicine 114, 154748. 10.1016/j.phymed.2023.154748 36933519

[B181] Xing W. Yang L. Peng Y. Wang Q. Gao M. Yang M. (2017). Ginsenoside Rg3 attenuates sepsis-induced injury and mitochondrial dysfunction in liver via AMPK-mediated autophagy flux. Biosci. Rep. 37 (4), BSR20170934. 10.1042/BSR20170934 28779013 PMC5577177

[B182] Xiong C. Guan F. Feng J. Jia J. Zhang J. Tu R. (2025). Prolonged sepsis triggers abnormal mitochondrial dynamics in the limb muscles and diaphragm. Mol. Cell Biochem. 480 (11), 5721–5739. 10.1007/s11010-025-05338-4 40591175

[B183] Xu C. Wang H. Wang H. Man J. Deng Y. Li Y. (2025). Schisandrin B regulates mitochondrial dynamics via AKT1 activation and mitochondrial targeting to ameliorate renal ischemia-reperfusion injury. Phytomedicine 141, 156672. 10.1016/j.phymed.2025.156672 40220406

[B184] Xu T. Xu H. Zhang J. Chen G. Han X. Zhang G. (2025). Isoferulic acid mitigates acute lung injury induced by sepsis through the inhibition of JAK2. Phytother. Res. 39 (7), 3225–3240. 10.1002/ptr.8526 40485414

[B185] Xu Q. Tan B. Lin P. Wang Y. Huang D. Yang X. (2026). Salvianolic acid C protects against sepsis-associated acute kidney injury through promotion of PGC1α-mediated renal gluconeogenesis. Phytomedicine 151, 157796. 10.1016/j.phymed.2026.157796 41539097

[B186] Yamano K. Youle R. J. (2013). PINK1 is degraded through the N-end rule pathway. Autophagy 9 (11), 1758–1769. 10.4161/auto.24633 24121706 PMC4028335

[B187] Yang Y. I. Woo J. H. Seo Y. J. Lee K. T. Lim Y. Choi J. H. (2016). Protective effect of brown alga phlorotannins against hyper-inflammatory responses in lipopolysaccharide-induced sepsis models. J. Agric. Food Chem. 64 (3), 570–578. 10.1021/acs.jafc.5b04482 26730445

[B188] Yang P. X. Fan X. X. Liu M. X. Zhang X. Z. Cao L. Wang Z. Z. (2024). Longxuetongluo capsule alleviate ischemia/reperfusion induced cardiomyocyte apoptosis through modulating oxidative stress and mitochondrial dysfunction. Phytomedicine 134, 155993. 10.1016/j.phymed.2024.155993 39244943

[B189] Yang T. T. Zhou L. H. Gu L. F. Qian L. L. Bao Y. L. Jing P. (2025). CHK1 attenuates cardiac dysfunction via suppressing SIRT1-ubiquitination. Metabolism 162, 156048. 10.1016/j.metabol.2024.156048 39454820

[B190] Yaribeygi H. Maleki M. Butler A. E. Jamialahmadi T. Sahebkar A. (2023). Sodium-glucose cotransporter 2 inhibitors and mitochondrial functions: state of the art. EXCLI J. 22, 53–66. 10.17179/excli2022-5482 PMC9939776 36814854

[B191] Yasuda M. Han J. W. Dionne C. A. Boyd J. M. Chinnadurai G. (1999). BNIP3alpha: a human homolog of mitochondrial proapoptotic protein BNIP3. Cancer Res. 59 (3), 533–537. 9973195

[B192] Ye L. Fu X. Li Q. (2025). Mitochondrial quality control in health and disease. MedComm 6 (8), e70319. 40821693 10.1002/mco2.70319 PMC12356995

[B193] You L. Wang M. Liu X. Song M. Zhou J. Feng J. (2025). DRP1: shedding light on the complex nexus of mitochondrial fission and breast cancer. Future Oncol. 21 (5), 593–603. 10.1080/14796694.2024.2447813 39936355 PMC11845117

[B194] Yu R. Liu T. Ning C. Tan F. Jin S. B. Lendahl U. (2019). The phosphorylation status of Ser-637 in dynamin-related protein 1 (DRP1) does not determine DRP1 recruitment to mitochondria. J. Biol. Chem. 294 (46), 17262–17277. 10.1074/jbc.RA119.008202 31533986 PMC6873174

[B195] Yu J. Lu Z. Chen B. He X. Zhao W. Cao H. (2024). Liang-Ge-San protects against viral infection-induced acute lung injury through inhibiting α7nAChR-mediated mitophagy. Phytomedicine 135, 156231. 10.1016/j.phymed.2024.156231 39566410

[B196] Zacharioudakis E. Agianian B. Kumar M. V. V. Biris N. Garner T. P. Rabinovich-Nikitin I. (2022). Modulating mitofusins to control mitochondrial function and signaling. Nat. Commun. 13, 3775. 10.1038/s41467-022-31324-1 PMC9262907 35798717

[B197] Zhang H. Yu F. Tian Z. Jia D. (2025a). Cardiolipin remodeling in cardiovascular diseases: implication for mitochondrial dysfunction. Acta Physiol. (Oxf) 241 (7), e70073. 10.1111/apha.70073 40530586

[B198] Zhang J. Ney P. A. (2009). Role of BNIP3 and nix in cell death, autophagy, and mitophagy. Cell Death Differ. 16 (7), 939–946. 10.1038/cdd.2009.16 19229244 PMC2768230

[B199] Zhang Y. Liu X. Bai J. Tian X. Zhao X. Liu W. (2016). Mitoguardin regulates mitochondrial fusion through MitoPLD and is required for neuronal homeostasis. Mol. Cell 61 (1), 111–124. 10.1016/j.molcel.2015.11.017 26711011

[B200] Zhang H. Xiao X. Pan Z. Dokudovskaya S. (2025b). mTOR signaling networks: mechanistic insights and translational frontiers in disease therapeutics. Signal Transduct. Target Ther. 10 (1), 428. 10.1038/s41392-025-02493-4 41469379 PMC12753737

[B201] Zhang J. Zhu X. Li Y. Wu Y. Du Y. Yang H. (2025). Parthenolide improves sepsis-induced coagulopathy by inhibiting mitochondrial-mediated apoptosis in vascular endothelial cells through BRD4/BCL-xL pathway. J. Transl. Med. 23, 80. 10.1186/s12967-025-06114-0 39825405 PMC11740428

[B202] Zhang Y. Ye Y. Feng Y. Li X. Chen L. Zou X. (2025). Kirenol alleviates cerebral ischemia-reperfusion injury by reducing oxidative stress and ameliorating mitochondrial dysfunction via activating the CK2/AKT pathway. Free Radic. Biol. Med. 232, 353–366. 10.1016/j.freeradbiomed.2025.03.022 40090600

[B203] Zhang W. Zhu W. Liu X. Yuan S. Li D. Huang H. (2025). Gypenoside XLIX targets SIRT1 to block YAP-NLRP3 activation and improve sepsis-induced cardiomyopathy. Phytomedicine 149, 157527. 10.1016/j.phymed.2025.157527 41265259

[B204] Zhang Y. Shen X. Shen Y. Wang C. Yu C. Han J. (2026). Cytosolic acetyl-coenzyme A is a signaling metabolite to control mitophagy. Nature 649 (8098), 1022–1031. 10.1038/s41586-025-09745-x 41225001 PMC12823391

[B205] Zhang R. Wu J. Wang D. Li K. Hou Y. Yu Y. (2026). Xin-Ji-Er-Kang alleviates chronic heart failure by suppressing mtDNA/cGAS-STING signaling through NR3C1-mediated MFN2 upregulation. Phytomedicine 150, 157714. 10.1016/j.phymed.2025.157714 41422729

[B63] Zheng W. Yan Q. Ni Y. Zhan S. Yang L Zhuang H. (2020). Examining the effector mechanisms of Xuebijing injection on COVID-19 based on network pharmacology. BioData Min. 13, 17. 10.1186/s13040-020-00227-6 33082858 PMC7563914

[B206] Zhong L. Lu M. Fang H. Li C. Qu H. Ding G. (2025). Secondary metabolites from Rehmannia glutinosa protect mitochondrial function in LPS-injured endothelial cells. Pharm. (Basel) 18 (8), 1125. 10.3390/ph18081125 PMC12389007 40872516

[B207] Zhou H. Toan S. (2020). Pathological roles of mitochondrial oxidative stress and mitochondrial dynamics in cardiac microvascular ischemia/reperfusion injury. Biomolecules 10 (1), 85. 10.3390/biom10010085 31948043 PMC7023463

[B208] Zhou Z. Y. Zhao W. R. Zhang J. Chen X. L. Tang J. Y. (2019). Sodium tanshinone IIA sulfonate: a review of pharmacological activity and pharmacokinetics. Biomed. & Pharmacother. 118, 109362. 10.1016/j.biopha.2019.109362 31545252

[B209] Zhou Y. Zhao Q. Zhang Y. Di L. Xue F. Xu W. (2024). A new andrographolide derivative ADA targeting SIRT3-FOXO3a signaling mitigates cognitive impairment by activating mitophagy and inhibiting neuroinflammation in Apoe4 mice. Phytomedicine 124, 155298. 10.1016/j.phymed.2023.155298 38185066

[B210] Zhu C. L. Yao R. Q. Li L. X. Li P. Xie J. Wang J. F. (2021). Mechanism of mitophagy and its role in sepsis induced organ dysfunction: a review. Front. Cell Dev. Biol. 9, 664896. 10.3389/fcell.2021.664896 34164394 PMC8215549

[B211] Zhuang N. Li L. Chen S. Wang T. (2016). PINK1-dependent phosphorylation of PINK1 and Parkin is essential for mitochondrial quality control. Cell Death Dis. 7 (12), e2501. 10.1038/cddis.2016.396 27906179 PMC5261015

